# Discovery and Application of Postnatal Nucleus Pulposus Progenitors Essential for Intervertebral Disc Homeostasis and Degeneration

**DOI:** 10.1002/advs.202104888

**Published:** 2022-02-23

**Authors:** Bo Gao, Bo Jiang, Wenhui Xing, Zaiqi Xie, Zhuojing Luo, Weiguo Zou

**Affiliations:** ^1^ State Key Laboratory of Cell Biology CAS Center for Excellence in Molecular Cell Sciences Shanghai Institute of Biochemistry and Cell Biology Chinese Academy of Sciences University of Chinese Academy of Sciences Shanghai China; ^2^ Institute of Orthopaedic Surgery Xijing Hospital Air Force Military Medical University Xi'an Shaanxi China; ^3^ Institute of Microsurgery on Extremities Shanghai Jiao Tong University Affiliated Sixth People's Hospital Shanghai 200233 China

**Keywords:** intervertebral disc, lineage tracing, nucleus pulposus cell atlas, Sc‐RNA seq, stem cell therapy

## Abstract

Intervertebral disc degeneration (IDD) results from the dysfunction of nucleus pulposus (NP) cells and the exhaustion of NP progenitors (ProNPs). The cellular applications of NP cells during IDD are currently limited due to the lack of in vivo studies showing whether NP cells are heterogeneous and contain ProNPs throughout postnatal stages. In this study, single‐cell RNA sequencing of purified NP cells is used to map four molecularly defined populations and urotensin II receptor (UTS2R)‐expressing postnatal ProNPs is identified, which are markedly exhausted during IDD, in mouse and human specimens. The lineage tracing shows that UTS2R^+^ ProNPs preferentially resides in the NP periphery with its niche factor tenascin‐C and give rise to functional NP cells. It is also demonstrated that transplanting UTS2R^+^ ProNPs with tenascin‐C into injured intervertebral discs attenuate the progression of IDD. The study provides a novel NP cell atlas, identified resident ProNPs with regenerative potential, and revealed promising diagnostic and therapeutic targets for IDD.

## Graphical abstract

The first single‐cell RNA sequencing of murine purified nucleus pulposus cells and the first in vivo evidence of postnatal resident ProNPs are provided with the validation of its potential niche (Tenascin‐C), self‐proliferative capacity, regenerative potential, and the function in attenuating Intervertebral disc degeneration progression. Also, the dynamic coupling role of RegNPs and HomNPs in modulating intervertebral disc homeostasis is discovered.

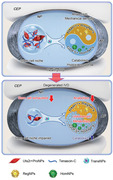

## 1 Introduction

Intervertebral disc degeneration (IDD) is the most common cause of back pain; it is a wide‐ranging clinical issue that imposes a huge socioeconomic burden.^[^ ^1^, ^2^ ^]^ IDD includes a wide range of degenerative pathologies associated with various clinical symptoms, including paresthesia,^[^ ^3^ ^]^ weakness of extremities,^[^ ^4^ ^]^ and back pain.^[^ ^5^ ^]^ It is characterized by abnormal synthesis and irregular distribution of extracellular matrix, disruption of the hypoxic environment, inflammation, and ingrowth of nociceptive nerves and blood vessels into the typically aneural and avascular tissues.^[^ ^6^ ^]^ The intervertebral disc (IVD) itself is composed of the nucleus pulposus (NP), annulus fibrosus (AF), and hyaline cartilaginous endplates (CEPs). IVD homeostasis largely relies on the “smooth operation” of NP cells, especially their maintenance and extracellular matrix (ECM) secretion. Several studies have shown that the dysfunction of NP cells and the exhaustion of notochord cells (NCs) are the primary causes that trigger the onset and progression of IDD.^[^ ^7^, ^8^, ^9^ ^]^ However, the cellular mechanisms underlying these processes are poorly defined. The potential roles of NP stem/progenitor cells are also unclear, as the origin, heterogeneity, and function of NP cells have not been systematically characterized so far. The current treatments for IDD and its induced herniation can be categorized as operative or conservative, with the latter primarily relying on anti‐inflammatory, muscle‐relaxant, and neurotrophic drugs. Cellular treatment has advanced in recent decades via the application of ADSCs, BMSCs, and UCBSCs, which have been shown to attenuate IDD progression and promote ECM remodeling.^[^ ^10^, ^11^, ^12^ ^]^ However, the harsh microenvironment of the IVD, which is hypoxic and avascular, still presents a challenge for the application of exogenous cells. This has prompted the identification of resident NP progenitors (ProNPs) as a potential cellular therapy.

NP cells have typically been described as a homozygous cell population derived from embryonic NCs. However, data from multi‐level sequencing technologies and subsequent lineage‐tracing animal models have raised the possibility that NP cells are a heterogeneous population. Our previous study indicated the heterogeneity of murine NP cells via fate mapping leptin receptor–positive (LepR+) cells.^[^ ^13^ ^]^ Another decent study identified two novel NP progenitor cell markers, disialoganglioside 2 (GD2) and tyrosine kinase receptor (Tie2), and found that Tie2^+^GD2^+^ cells possessed stem cell properties with self‐renewal potential. These cells were thought to differentiate into Tie2^−^/GD2^−^ cells, providing evidence that the NP harbored its own resident progenitor cells.^[^ ^14^ ^]^ However, the potential heterogeneity of NP cells and their representative cellular functions remain ill‐defined due to the lack of established markers (particularly membrane proteins) to identify, isolate, and modulate these cells. In addition, the anatomical positions of the adjacent NP, AF, and CEP make it extremely difficult to solely analyze NP cells without contamination from other nearby cells. These issues inevitably affect the clarification of NP cell population ontogeny and function during disc homeostasis or degeneration. Therefore, single‐cell analysis of NP cells and lineage tracing of the potential purified NP cells in vivo via specific markers are required to validate the existence of postnatal ProNPs.

Identifying ProNPs and functional NP cells can increase our understanding of spinal homeostasis and provide new avenues for the therapeutic treatment of IDD. In this study, we performed scRNA‐seq analysis to produce a cell atlas using purified NP cells along with their representative cellular functions. This is the first such map made for the genetic mice model based purified NPs. Importantly, we identified urotensin II receptor–expressing (UTS2R^+^) NP cells as the postnatal resident stem/progenitor cells and validated the application of ProNPs, combined with ECM specific to their niche, as a potential therapy for IDD.

## 2 Results

### 2.1 Single‐Cell Sequencing Analysis Revealed Four Subpopulations of Murine NP Cells

**Figure 1:**
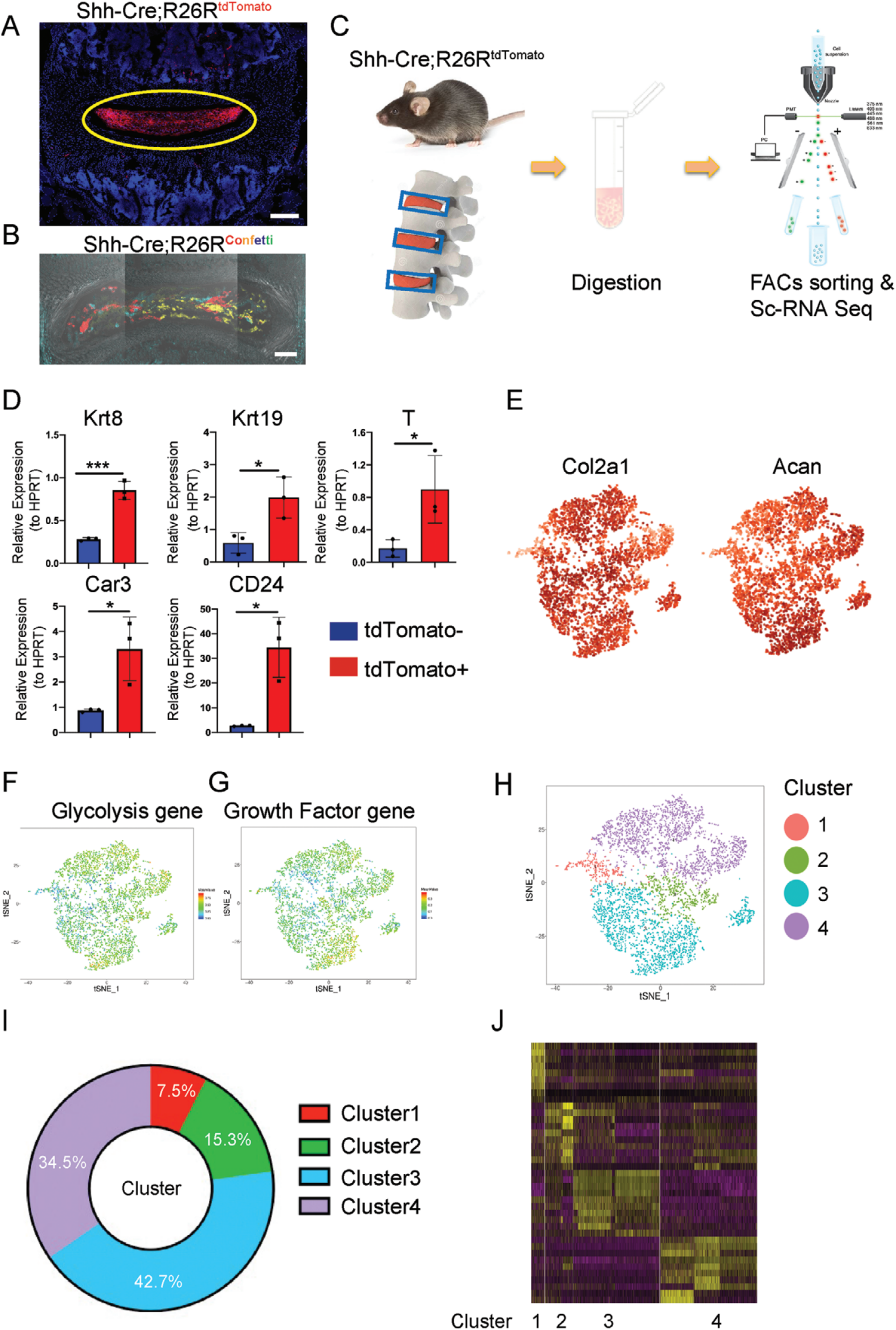
Single‐cell RNA sequencing analysis of murine NP cells from Shh‐Cre;R26R^tdTomato^ mice. A) Representative images of lumbar sections from 4‐week‐old Shh‐Cre;R26R^tdTomato^ mice. The yellow circle shows NP tissue. Scale bars, 100 µm. B) Representative image of lumbar sections from 4‐week‐old Shh‐Cre; R26R^confetti^ mice. Scale bars, 100 µm. C) Schematic workflow of the experimental strategy. Purified NP cells were isolated from Shh‐Cre;R26R^tdTomato^ mice, enzymatically digested, and FACS‐sorted to isolate tdTomato^+^ cells, which then underwent single‐cell RNA sequencing analysis via BD Rhapsody. D) qRT‐PCR of expression of NP marker genes, including *Krt8*, *Krt19*, *T*, *Car3*, and *CD24* related to HPRT in Shh‐Cre;R26R^tdTomato+^ and Shh‐Cre;R26R^tdTomato‐^ cells. E) Dot plots showing the expression of *Col2a1* and *Acan* within the t‐SNE map. F,G) t‐SNE plots of glycolysis gene (F) and growth factor gene (G) distribution. H) Representative image of t‐SNE analysis showing the four clusters of Shh‐Cre;Ai9^+^ NP cells. (I) Relative percentage of each cluster among Shh‐Cre;Ai9 NP cells. J) Heatmap revealing the scaled expression of differentially expressed genes for each cluster. *n* = 3. Data are presented as mean ± standard deviation. ^*^ *P* < 0.05, ^**^ *P* < 0.01; N.S., not significant as determined by two‐tailed Student *t* tests.

The difficulty of isolating NP tissue from AF tissue in mice hinders the characterization and understanding of NP cells and their subpopulations, functions, and intrinsic interactions. In this study, we isolated highly purified murine NP cells by crossing Shh‐Cre mice with Rosa26‐tdTomato or Rosa26‐Confetti reporter mice to generate Shh‐Cre;Rosa26^tdTomato^ transgenic mice (here referred to as Shh‐Cre;Ai9) or Shh‐Cre;Rosa26^confetti^ mice. This genotype efficiently mediated Cre recombinase‐driven tdTomato or Confetti expression in embryonic notochord‐derived cells without labeling AF cells (**Figure** **1**A,B). FACS‐sorted NP cells from Shh‐Cre;Ai9 mice showed a clear tdTomato signal while Ai9^+^ cells from control littermates did not, indicating no leakage of tdTomato into the surrounding disc tissues (Figure 1C and Figure S1A: Supporting Information). The qRT‐PCR analysis of Shh‐Ai9^+^ NP cells found significantly higher expression of reported NP genes, such as *Krt8*, *Krt19*, *T*, *Car3*, and *CD24*, validating the FACS‐sorted NP cells (Figure 1D). Next, 4910 Shh‐Cre;Ai9^+^ single NP cells were harvested from 1‐month‐old Shh‐Cre;Ai9 mice and analyzed with a BD Rhapsody single‐cell RNA sequencing platform. Nearly all cells expressed tdTomato (Figure S1B, Supporting Information), further validating the efficacy of the FACS sorting system. Of note, the scRNA‐seq analysis showed that the core genes encoding anabolic metabolism, *Col2a1* and *Acan*, were generally expressed among NP cells (Figure 1E). NP tissue is an avascular, relatively hypoxic, and self‐modulated homeostatic tissue; hence, the genes related to glycolysis and growth factors were generally highly expressed in these cells (Figure 1F,G). A t‐SNE plot from the scRNA‐seq analysis displayed four distinct subpopulations (Figure 1H), with Clusters 1, 2, 3, and 4 representing ≈7.5%, 15.3%, 42.7%, and 34.5% of all Shh‐Cre;Ai9^+^ NP cells, respectively (Figure 1I). Each cluster displayed a different gene expression pattern (Figure 1J). The KEGG analysis revealed that Cluster 1 was mainly enriched in cell adhesion and “TGF‐beta signaling pathway” (Figure S1C–E: Supporting Information). Cluster 2 possessed few specific functions such as “Folate biosynthesis” and “PI3K‐Akt signaling pathway” (Figure S1C–E: Supporting Information). Cluster 3 specifically included several metabolic/biosynthetic processes, such as citric acid (TCA) cycle, glycosaminoglycan biosynthesis, sulfur metabolism, biosynthesis of unsaturated fatty acids, and involved many kinds of signaling pathway genes such as “Hippo, Wnt, VEGF, Thyroid hormone, Estrogen, Notch, Toll‐like receptor” (Figure S1C–E: Supporting Information). Cluster 4 was specifically enriched in functions such as “anti‐inflammatory gene,” “glycolysis gene,” “glycosphingolipid biosynthesis,” and “protein digestion and absorption” (Figure S1C–E: Supporting Information). These data revealed that murine NP cells consisted of heterogeneous subpopulations, and provided us new insights into the functions, gene expression levels, ECM modulation, and metabolic homeostasis of these different subpopulations.

### 2.2 Characterization of Functional NP Clusters

**Figure 2:**
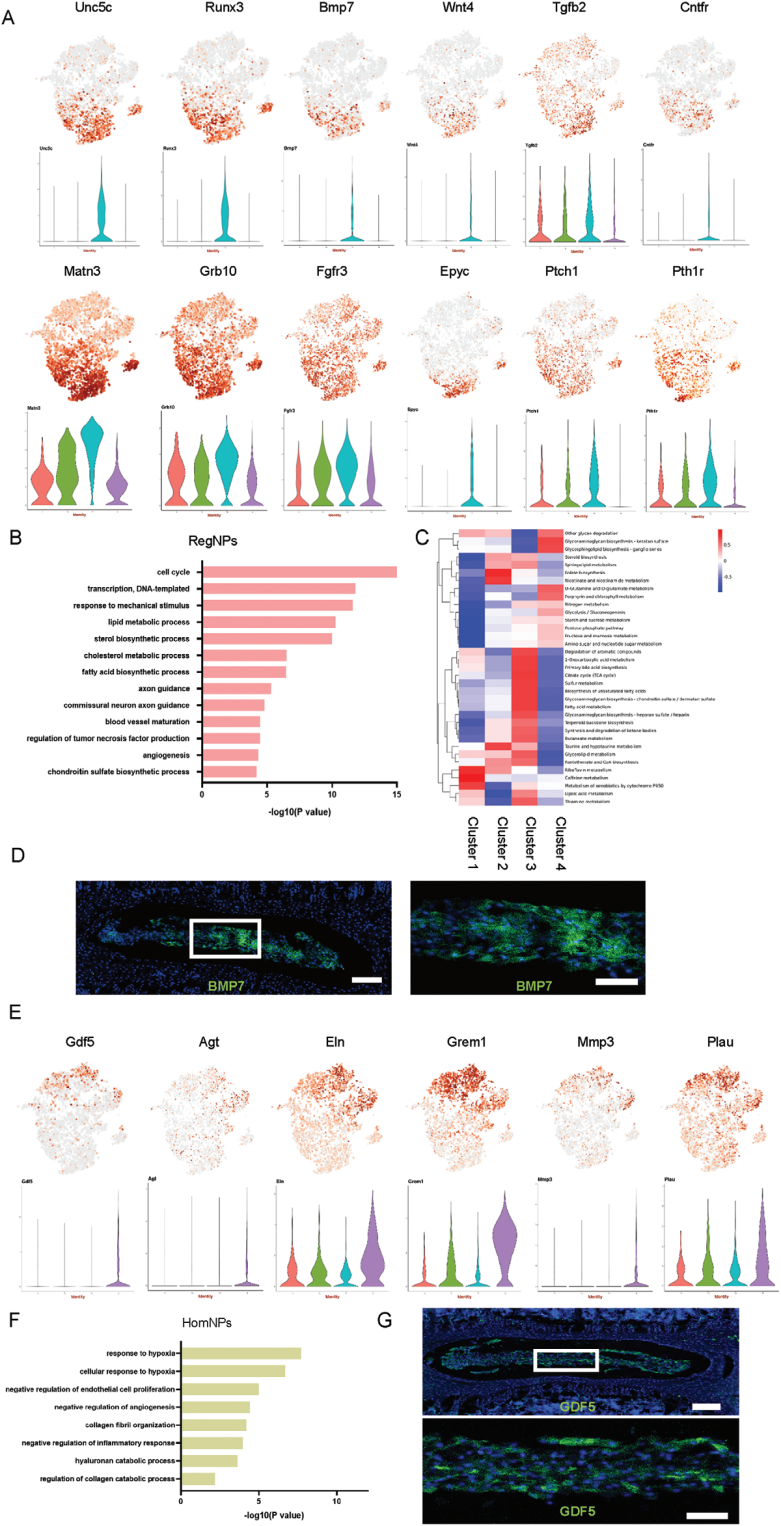
RegNPs were a metabolically active and mechanically sensitive population, while HomNPs were sensitive to hypoxia with “degenerative” potential. A) t‐SNE plots and representative violin plots showing the expression of Unc5c, Runx3, Bmp7, Wnt4, Tgfb2, CNTFR, Matn3, Grb10, Fgfr3, Epyc, Ptch1, and Pth1r on the t‐SNE map. B) Representation analysis of GO categories showing different functions for RegNPs. C) Heatmap revealing metabolic‐related functions and pathways for RegNPs. D) Representative images of lumbar spine sections from 4‐week‐old wild‐type mice stained for BMP7. Scale bars, 100 µm (*n* = 3 mice per group). E) t‐SNE plots and representative violin plots showing the expression of Gdf5, Agt, Eln, Grem1, Mmp3, and Plau on the t‐SNE map. F) Representative analysis of GO categories showing different functions for HomNPs. G) Representative images of lumbar spine sections from 4‐week‐old WT mice stained for GDF5. The lower image shows a high‐magnification view of the indicated area from the upper image. Scale bars, 100 µm (*n* = 3 mice per group).

Clusters 3 and 4 together contained 77.2% of all NP cells. Therefore, we first checked their enriched functions and investigated whether they represented the potential functional NP cells. The characteristic genes of Cluster 3 were enriched in the functions of cellular response to PTH (Pth1r) and mechanical stimulus (Ptch1), cartilage development (Bmp7, Wnt4, Matn3, Fgfr3, and Epyc), axon guidance and neuron activation (CNTFR, Unc5c, and Runx3), and angiogenesis (Tgfb2 and Grb10) (**Figure** **2**A). Consistent with these characteristic gene functions, the GO analysis showed that Cluster 3 was enriched in terms associated with cell cycle, mechano‐sensing, cytokine production, and metabolic/biosynthetic processes including “lipid metabolic process,” “sterol biosynthetic process,” “cholesterol metabolic process,” “fatty acid biosynthetic process,” and “chondroitin sulfate biosynthetic process” (Figure 2B). We performed a QuSAGE gene expression analysis to further characterize the metabolic properties of Cluster 3, finding that Cluster 3 was involved in several significant metabolic processes, including the TCA cycle, fatty acid metabolism, sulfur metabolism, and glycosaminoglycan biosynthesis (Figure 2C). As NP is an avascular, non‐innervated, and immune silent tissue, the high enrichment of genes related to axon guidance, angiogenesis, and TNF production indicated that Cluster 3 might be the “chief culprit” for the onset of disc degeneration and the inflammatory cascade. The immunostaining of murine disc samples confirmed the BMP7^+^ NP subpopulations (Figure 2D). We also harvested human NP specimens of different degeneration grades (modified Pfirrmann, Grades II and V) and immune‐stained them for CNTFR, another membrane marker specifically expressed in Cluster 3. The result showed that the percentage of CNTFR^+^ human NP cells was significantly lower in Grade V disc specimens compared with Grade II (Figure S2A,B: Supporting Information). We employed cell communication analysis to further clarify the interactions and functions of NP cells in Cluster 3, finding that Cluster 3 mainly modulated the other three clusters via the secretion of collagens including COL2a1, COL9a1, COL11a1, and BMP7 (Figure S3A, Supporting Information). In return, Cluster 3 could be modulated mainly via receptors such as Integrin *α*1*β*1, NRP1, and FGFR3 (Figure S3B, Supporting Information). We further analyzed the specific transcription factors (TFs) of Cluster 3, screened the downstream targets co‐modulated by at least three specific TFs, and analyzed their enriched functions (Figure S3C, Supporting Information). scRNA‐seq analysis revealed specific TFs associated with Cluster 3 (Hadc2, Gata2, Egr4, En1, Msx2, Rel, Jun, and Lef1). GO analysis showed that Cluster 3 mainly focused on “cell cycle,” “mechanical stimulus,” and several biosynthetic processes including lipid and peptide metabolism (Figure S3C–E: Supporting Information), further confirming the characteristics of Cluster 3 NP cells. Therefore, we dubbed the Cluster 3 NP cells “Regulatory NP cells” or “RegNPs.”

The diverse functions of RegNPs led us further divided it into 3 subpopulations‐RegNPs‐A, RegNPs‐B and RegNPs‐C (Figure S4A, Supporting Information) . The angiogenesis, secretion protein, growth factor and SASP related genes were differentially distributed among these 3 subpopulations (Figure S4B–E: Supporting Information). RegNPs‐A expressed higher expression of Per1 (circadian rhythm), Apoe (cholesterol homeostasis), Ecm2 (collagen binding) and Bhlhe41 (circadian rhythm). RegNPs‐B expressed Ngf (innervation), Vegfa (angiogenesis), Irx1 and Nfkb1 (inflammatory response). RegNPs‐C expressed Ncam1 (regulation of sensory perception of pain), Pcna (cell proliferation), Dag1 (cellular response to cholesterol) and Ezh2 (senescence) (Figure S4F–H: Supporting Information). GO and KEGG analysis indicated that RegNPs‐A is enriched in the function such as “positive regulation of “response to mechanical stimulus”, “ECM‐receptor interaction” and “circadian rhythm modulation”. RegNPs‐B in “sterol biosynthetic process” and “angiogenesis”. RegNPs‐C is enriched primarily in “cell cycle” and “TCA cycle” (Figure S5A–C: Supporting Information).

The characteristic genes of Cluster 4 were enriched in collagen catabolism (Mmp3), hypoxic response (Plau), fibrosis (Gdf5, Agt, and Grem1), and ECM organization (Eln) (Figure 2E). This indicated that Cluster 4 might be involved in catabolic metabolism and fibrosis, which are the typical outcomes of IDD. Accordingly, the GO analysis showed that Cluster 4 NP cells were mainly involved in hypoxic response, hyaluronan and collagen catabolism, and negative regulation of endothelial cell proliferation, angiogenesis, and inflammation (Figure 2F). Importantly, the pro‐metabolic functions of RegNPs coupled with the pro‐catabolic characteristics of Cluster 4 might contribute to the constant remodeling of the ECM in NP tissue. The immunostaining of murine disc samples and human IVD specimens of different degeneration grades (modified Pfirrmann, Grades II and V) confirmed that GDF5^+^ NP cells resided in NP tissue (Figure 2G). Also, interestingly, the percentage of human GDF5^+^ NP cells was significantly higher in Grade V disc specimens than in Grade II specimens (Figure S2C,D: Supporting Information). The cell communication analysis showed that Cluster 4 modulated RegNPs by secreting CD44, Efna1, and Efnb2. Cluster 4 NP cells could also be modulated mainly by RegNPs via receptors such as Integrin *α*10*β*1 and *α*1*β*1 complex (Figure S6A,B: Supporting Information). The scRNA‐seq analysis revealed several specific TFs associated with Cluster 4 (Cebpb, Elf1, Foxo1, Foxq1, Nfkb1, Snail1, Pou3f1, and Hivep1) (Figure S6C, Supporting Information). Further, the GO analysis showed that Cluster 4 was mainly involved in hypoxic response and protein catabolism (Figure S6D,E: Supporting Information), further indicating that Cluster 4 cells sensed the local environment of the NP and worked together with RegNPs to maintain NP homeostasis. Therefore, we named Cluster 4 as the “Homeostatic NP cells” or “HomNPs.”

HomNPs contained 3 subpopulations with diverse functions, and genes encoding secretion proteins and growth factors were differentially distributed among HomNPs (Figure S7A–C, Supporting Information). tSNE analysis showed that Mmp2 (collagen catabolic process), Adamts2 (collagen catabolic process), and Itgbl1 (regulator of fibrogenesis) highly expressed in HomNPs‐A. Tgfbr3 (response to hypoxia), Bmp2 (cellular response to BMP stimulus, response to hypoxia) and Cytl1(chondroitin sulfate proteoglycan biosynthetic process) preferentially expressed in HomNPs‐B. Ghr (receptor signaling pathway via JAK‐STAT), Inhba (negative regulation of cell cycle), and Il11 (anti‐inflammation) preferentially expressed in HomNPs‐C (Figure S7D–F: Supporting Information). GO and pathway analysis showed that HomNPs‐A was mainly enriched in “collagen fibril organization”, HomNPs‐B was partially enriched in “extracellular matrix organization” and “negative regulation of endothelial cell proliferation”. HomNPs‐C was enriched in “response to hypoxia” (Figure S7G,H: Supporting Information).

**Figure 3:**
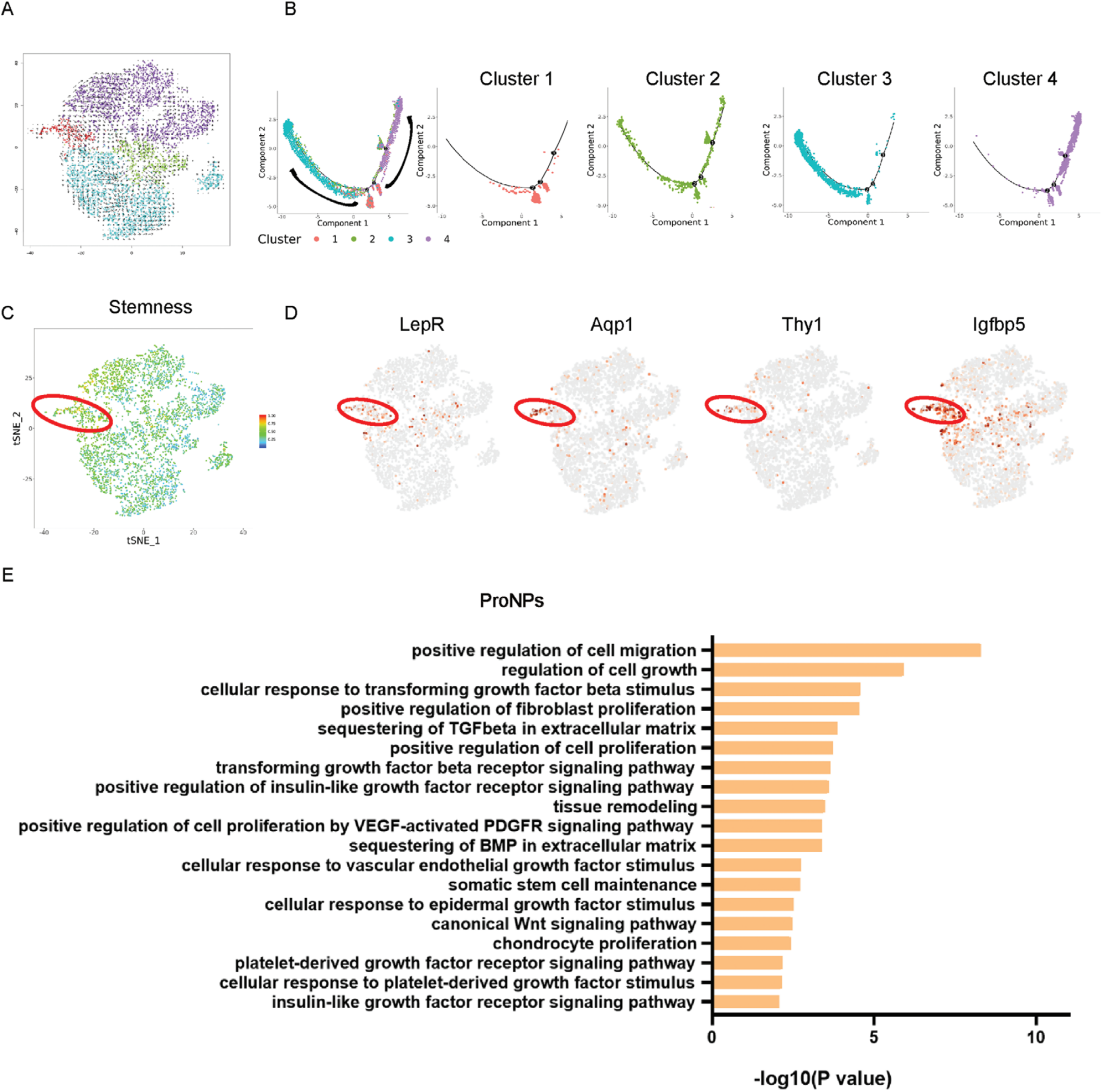
Characterization of potential NP cell progenitors in Cluster 1. A) Velocity image showing the potential trajectories among NP subpopulations. B) Monocle pseudospace trajectories revealing the NP cell lineage progression colored according to cell types. C) Stemness analysis of NP cell subpopulations using mesenchymal stem/progenitor cell markers. D) Dot plots showing the expression of LepR, Aqp1, Thy1, and Igfbp5 on the t‐SNE map. E) Representation analysis of GO categories showing different functions for ProNPs.

The velocity and pseudotime analysis provided the hint that Cluster 2 was seemingly located at a transit state and likely represented the transitional population of Clusters 3 and 4 (**Figure** **3**A,B). We therefore named Cluster 2 as the “Transient NP cells” or “TransNPs.” The t‐SNE gene expression analysis showed that Col4a5 (ECM organization), Omd (cell adhesion), Bmp3 (cartilage development), Frzb (Wnt‐protein binding), and Ibsp (cell adhesion) were highly expressed in TransNPs (Figure S8B, Supporting Information). The cell communication bubble plot analysis showed that TransNPs modulated the other clusters primarily via secreting COL2a1, FGFR3, and NOTCH2 (Figure S8C, Supporting Information). TransNPs could be modulated mainly by RegNPs via receptors such as Integrin *α*10*β*1, BMPR2, and the Ephb family (Figure S8D, Supporting Information). Next, the scRNA‐seq analysis identified several TFs specifically expressed by TransNPs (Irf8, Stat5a, Dbp, and Foxl1) (Figure S8E, Supporting Information). We further investigated by screening the downstream targets co‐modulated by at least two specific TFs and analyzing their enriched functions to clarify the function of TransNPs (Figure S8F, Supporting Information). Finally, the GO analysis revealed that TransNPs were mainly involved in cytokine response, glycoprotein biosynthesis, and mesenchymal cell development (Figure S8G, Supporting Information).

### 2.3 Characterization of ProNPs

It has long been debated whether NP tissue contains resident progenitor cells that show “stem‐like” properties and are able to differentiate into mature, functional NP cells, especially in the postnatal stage. The pseudotime and velocity analysis of postnatal NP cells from 1‐month‐old mice showed that Cluster 1 was located at the origin of the trajectory process, while RegNPs and HomNPs represented two different trajectory directions (Figure 3A,B: Supporting Information). We further performed “Stemness” analysis by evaluating the expression of several potential stem/progenitor cell markers in NP cells (the gene list is presented in Table S1: Supporting Information). The “stemness” genes were mainly enriched in the Cluster 1 population (Figure 3C). LepR, Aqp1, Thy1, and Igfbp5, specifically, were highly enriched in Cluster 1 (Figure 3D). Of note, our previous study showed that LepR‐Cre;Rosa26^tdTomato^ mice traced from embryonic stages labeled nearly 90% of NP cells, confirming that LepR^+^ NP cells contained NP cell progenitors.^[^ ^13^ ^]^ Therefore, we named Cluster 1 cells as “NP progenitor cells” or “ProNPs.” The GO analysis showed that ProNPs were enriched in “positive regulation of cell migration” and “regulation of cell growth” and highly responded to several signaling pathways involved in cell differentiation, development, and homeostasis (such as TGF‐beta, EGF, etc.) (Figure 3E). We further analyzed the specific TF of ProNPs (Hoxa9, Hoxd9, Etv5, Foxp2, Mecom, Nfatc4, Nr2f2, and Sox4) (Figure S9A, Supporting Information), screened the downstream targets, and analyzed their enriched functions (Figure S9B, Supporting Information). The GO analysis revealed that TFs in ProNPs were mainly involved in the regulation of cell growth and differentiation (Figure S9C, Supporting Information). These potential TFs, combined with specific markers, can provide potential therapeutic targets for modulating the ProNP subpopulation. These results validated that Cluster 1 NP cells harbored NP cell progenitors.

### 2.4 UTS2R was a Novel Cell‐Surface Marker of NP Progenitor Cells

**Figure 4:**
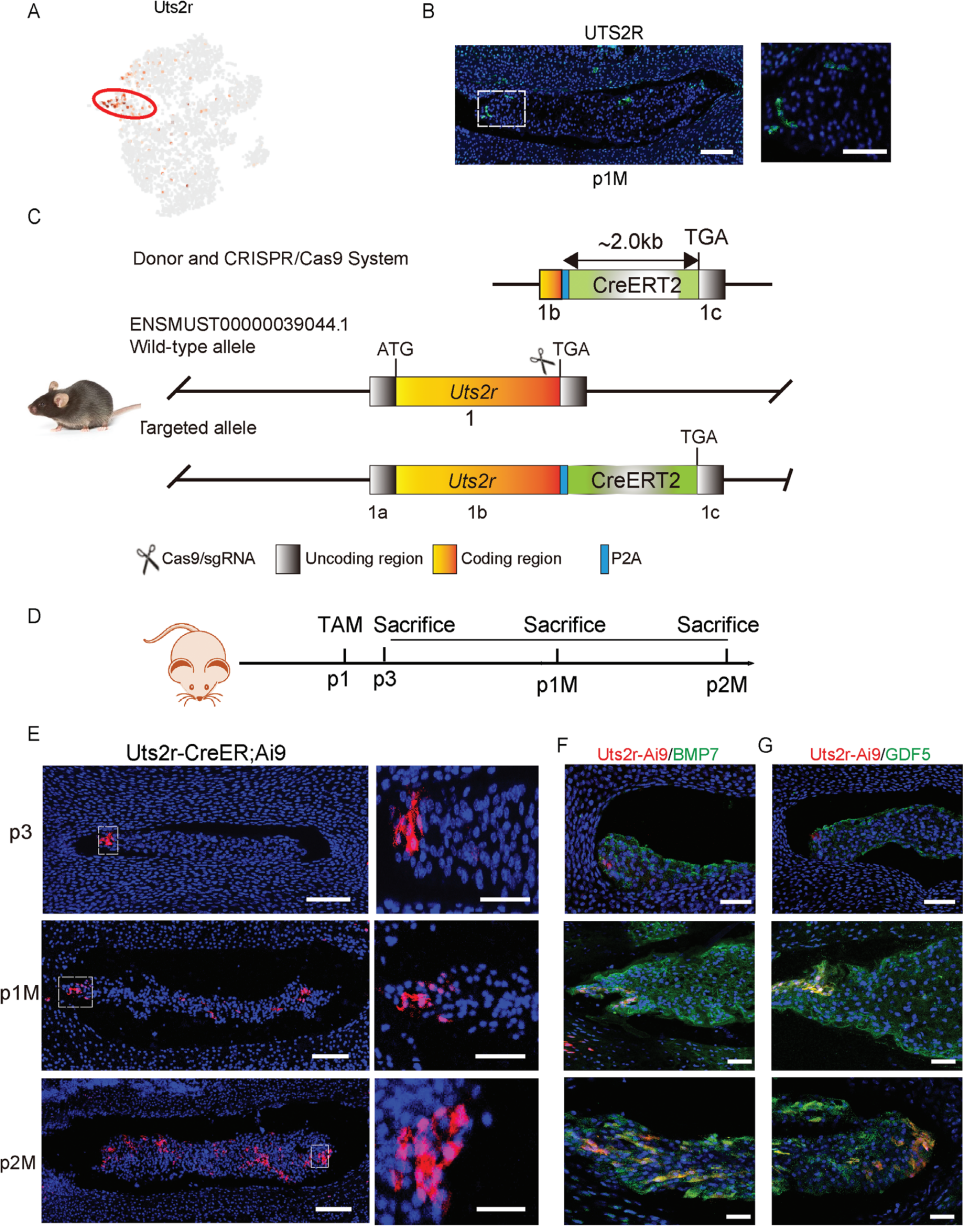
Lineage tracing of UTS2R^+^ NP cells. A) Dot plot showing the expression of Uts2r on the t‐SNE map. B) Representative immunofluorescence imaging of UTS2R (green) in postnatal 1‐month‐old WT mice. The right image shows high magnification of the indicated area from the left image (*n* = 3 mice per group). Scale bars, 100 µm. C) Construction strategy of Uts2r‐CreER transgenic mice using the CRISPR/Cas9 System. D) Diagram showing postnatal day 1 (P1) Uts2r‐CreER;Ai9^/+^ mice administered with one dosage tamoxifen and sacrificed at postnatal day 3 (P3), 1 month (P1M), or 2 months (P2M). E) Representative immunofluorescence imaging of Uts2r‐CreER;Ai9^+^ cells (red). The right images show high magnification of the indicated area from the left image (*n* = 3 mice per group). Scale bars, 100 µm. F,G) Representative immunofluorescence imaging of Uts2r‐CreER;Ai9^+^ cells (red), BMP7 (green) (F) or GDF5 (green) (G). The right images show high magnification of the indicated area from the left image (*n* = 3 mice per group). Scale bars, 100 µm.

The identification and application of stem cells via membrane markers are particularly important for pre‐clinical translation. Our gene expression analysis revealed that the urotensin II receptor (UTS2R, also known as GPR14) is a novel candidate marker of ProNPs (**Figure** **4**A). The immunofluorescence staining with an anti‐UTS2R antibody showed UTS2R^+^ NP cells residing in the peripheral region of NP tissue (Figure 4B). For fate mapping of postnatal UTS2R^+^ ProNPs, we constructed tamoxifen‐inducible Uts2r‐CreER mice (Figure 4C) and crossed them with Rosa26;tdTomato mice to generate Uts2r‐CreER;Rosa26^tdTomato^ mice (here referred to as Uts2r‐CreER;Ai9/^+^). The mice were injected with tamoxifen at P1, and the cells were traced for 48 h, 1 month, or 2 months (Figure 4D). Uts2r‐CreER;Ai9^+^ ProNPs were found to be distributed in the peripheral region of NP cells after 48 h (Figure 4E), and increasing numbers of Ai9^+^ cells were noted throughout the NP after 2 months (Figure 4E). The immunostaining of BMP7 (RegNPs) or GDF5 (HomNPs) in the IVD of Uts2r‐CreER;Ai9 mice displayed that ProNPs traced after 48 h from P1 had no overlap with RegNPs or HomNPs, while ProNPs gradually gave rise to functional NP cells after 1‐month and 2‐month fate mapping (Figure 4F,G). These results indicated the existence of postnatal ProNPs and identified Uts2r as a novel potential cell‐surface marker.

### 2.5 UTS2R^+^ ProNPs Displayed the Characteristics of Stem/Progenitor Cells

**Figure 5:**
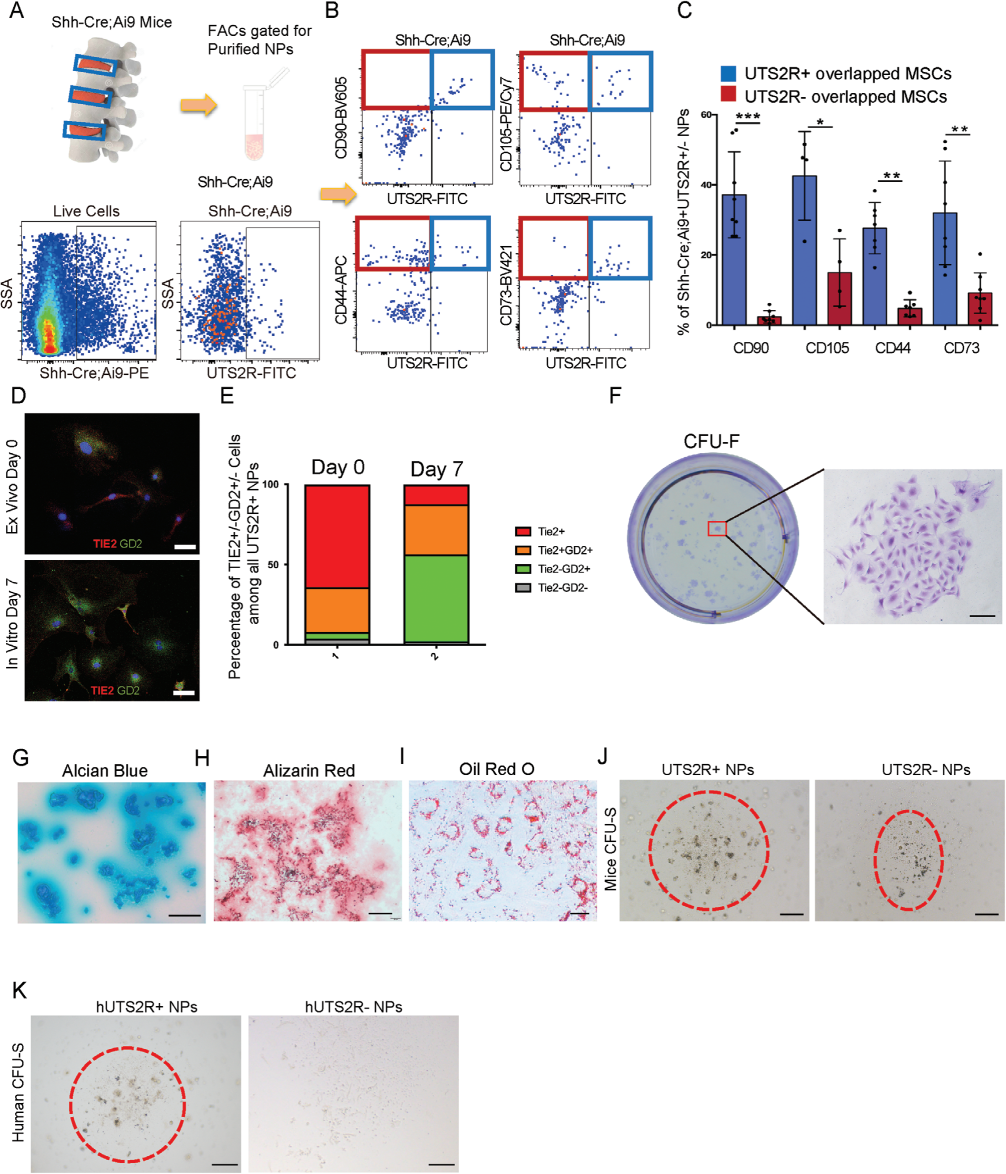
Identification of UTS2R^+^ NP cell progenitors in Cluster 1. A,B) Diagram of isolating (A) and FACS gating strategy of Shh‐Cre;Ai9^+^UTS2R^+^ ProNPs (B). C) Quantification of the percentage of ProNPs expressing markers of mesenchymal stem/progenitor cells in 4‐week‐old Shh‐Cre;Ai9 mice, *n* ≥ 4. D) Representative immunofluorescence staining of Tie2 and GD2 in murine UTS2R^+^ NP cells from the *ex vivo* plated group and the in vitro 7‐day cultured group. Cells were sorted by isolating disc cells from 4‐week‐old Shh‐Ai9 mice and gating on the Shh‐Ai9^+^UTS2R^+^ channel. E) Quantification of Tie2^+^, Tie2^+^GD2^+^, Tie2^−^GD2^+^, and Tie2^−^GD2^−^ cells in all sorted murine UTS2R NP cells. F) Crystal violet staining revealing the CFU‐F‐forming ability of rat UTS2R^+^ ProNPs. G–I) Alcian blue (G), Alizarin Red (H), and Oil Red O staining (I) of rat ProNPs under chondrogenic, osteogenic, or adipogenic differentiation conditions. J,K) CFU‐sphere‐forming ability of UTS2R^+^ and UTS2R^−^ NP cells isolated from rat (J) and human specimens (K). Data are presented as mean ± standard deviation. ^*^ *P* < 0.05, ^**^ *P* < 0.01; N.S., not significant as determined by ANOVA.

We sorted Ai9^+^UTS2R^+^ NP cells (to exclude contamination by AF cells) from 1‐month‐old Shh‐Cre;Ai9/^+^ mice via FACS to determine whether UTS2R^+^ ProNPs harbored potential stem/progenitor cells (**Figure** **5**A). FACs analysis showed that UTS2R^+^ ProNPs contained a much higher percentage of CD90^+^, CD105^+^, CD44^+^, and CD73^+^ cells (≈38%, 42%, 29%, and 33%, respectively) compared with UTS2R^−^ NP cells (Figure 5B,C). We further compared UTS2R^+^ NP cells with previously reported NP progenitors classified by Tie2 and GD2 expression.^[^ ^14^ ^]^ Our results showed that ≈64% of UTS2R^+^ NP cells were Tie2 single‐positive while 31% were Tie2/GD2 double‐positive, indicating that most UTS2R^+^ NP cells were NP progenitor cells (Figure 5D,E). After 7‐day in vitro culture, only 12% of UTS2R^+^ ProNPs‐derived cells were Tie2^+^, 31% were Tie2^+^GD2^+^, and 54% were Tie2^−^GD2^+^, indicating the gradual differentiation of ProNPs (Figure 5D,E). Because rat NP cells can be easily isolated in large numbers and without the contamination of AF cells, we harvested and FACS‐sorted rat UTS2R^+^ NP cells from lumbar and caudal IVD and investigated whether they displayed stem/progenitor cell potential similar to murine ProNPs. The result showed that rat UTS2R^+^ ProNPs formed CFU‐F and displayed chondrogenic, osteogenic, and adipogenic differentiation abilities in vitro (Figure 5F–I). Since NP cells reside in a 3D gel‐like microenvironment, we cultured rat UTS2R^+^ NP cells in Matrigel, in which these cells displayed better sphere‐forming ability than rat UTS2R^−^ NP cells (Figure 5J). We further harvested and sorted UTS2R^+/−^ NP cells from human NP specimens (Grade II) via FACS and cultured them in Matrigel to investigate whether human NP cells contained UTS2R^+^ NP cells with sphere forming ability. The results showed that human UTS2R^+^ NP cells also displayed markedly better sphere‐forming ability compared with UTS2R^−^ NP cells (Figure 5K). Together, these results indicated that UTS2R^+^ ProNPs displayed stem/progenitor cell properties and that UTS2R was a viable membrane marker for isolating ProNPs.

### 2.6 UTS2R^+^ ProNPs were Exhausted During IDD

**Figure 6:**
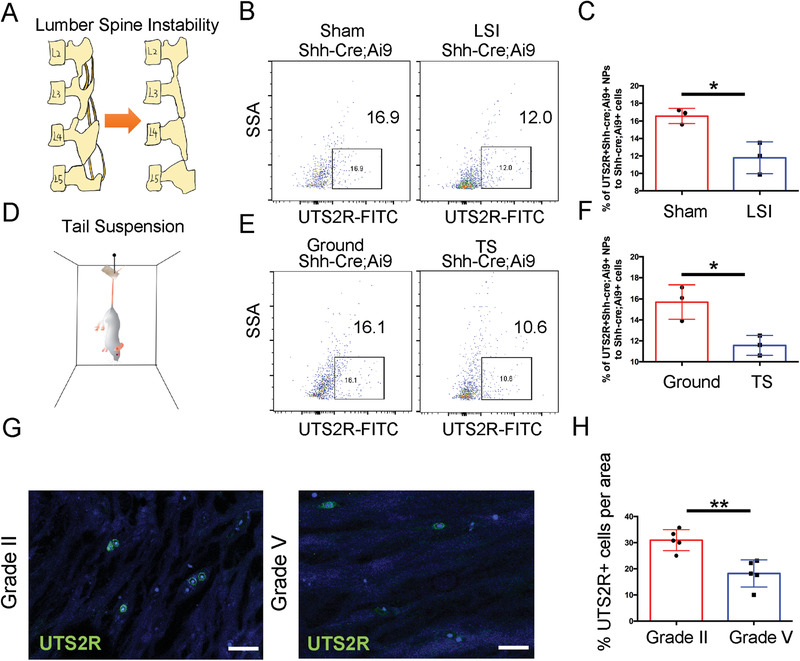
ProNPs were exhausted during intervertebral disc degeneration. A) LSI model was established in 4‐week‐old Shh‐Cre;Ai9 mice. B,C) FACS analysis and quantification of Shh‐Cre;Ai9^+^UTS2R^+^ NP cells in Shh‐Cre;Ai9 mice from Sham (control) and LSI groups. D) Tail suspension model was established in 4‐week‐old Shh‐Cre;Ai9 mice. E,F) FACS analysis and quantification of Shh‐Cre;Ai9^+^UTS2R^+^ NP cells in Shh‐Cre;Ai9 mice from Ground (control) and TS groups. G,H) Immunofluorescence staining and representative quantification data of UTS2R^+^ cells in human NP specimens at different degeneration levels. *n* = 3. Data are presented as mean ± standard deviation. ^*^ *P* < 0.05, ^**^ *P* < 0.01; N.S., not significant as determined by two‐tailed Student *t* tests.

We established two disc degeneration models [lumbar instability model (LSI) and tail suspension modal (TS)] in Shh‐Cre;Ai9 mice to investigate the outcome of ProNPs during IDD (**Figure** **6**A,D). The FACS analysis of lumbar disc cells showed that the percentage of Shh‐Cre;Ai9^+^UTS2R^+^ ProNPs was markedly reduced in the LSI‐induced IDD model than in the Sham control group (Figure 6B,C). The percentage of Shh‐Cre;Ai9^+^UTS2R^+^ ProNPs also significantly decreased in the TS‐induced IDD model than in the Ground control group (Figure 6E,F). Next, we aimed to elucidate the specificity of UTS2R^+^ cells in human NP specimens and investigate their correlation with IDD. We performed the microendoscopic discectomy of the NP with different degeneration grades and carefully collected the pure NP cells for immunostaining. Our results showed that the percentage of UTS2R^+^ cells was significantly lower in Grade V NP specimens than in Grade II specimens (Figure 6G,H). These findings indicated that UTS2R^+^ NP cells were exhausted during IDD, which indicate ProNPs might play essential roles in maintaining physiological disc homeostasis and modulating the progression of human IDD.

### 2.7 UTS2R^+^ ProNPs Attenuated IDD

**Figure 7:**
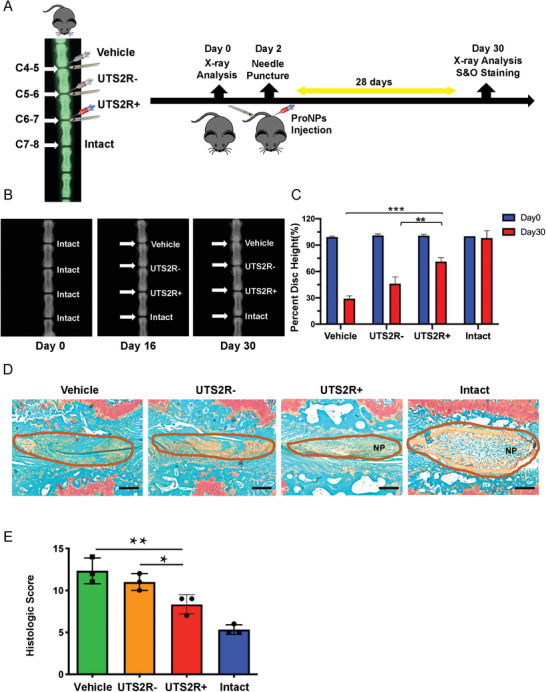
Transplantation of murine UTS2R^+^ ProNPs attenuated injury‐induced intervertebral disc degeneration. A) Diagram of the experimental outline displaying the time‐point and procedure of establishing caudal spine injury models and transplanting UTS2R^+/−^ NP cells. B,C) X‐ray scan and quantification revealing the caudal disc space and height between vehicle‐treated, UTS2R^−^ NP–transplanted, UTS2R^+^ NP–transplanted, and intact groups. UTS2R^+/−^ NP cells were FACS‐sorted from 1‐month‐old Shh‐Cre;Ai9 mice. D,E) Safranin O/Fast Green staining (D) and the Histologic Score (E) revealing the progress of injury‐induced IDD between vehicle‐treated, UTS2R^−^ NP–transplanted, UTS2R^+^ NP–transplanted, and intact groups. Scale bars, 100 µm. *n* = 3. Data are presented as mean ± standard deviation. ^*^ *P* < 0.05, ^**^ *P* < 0.01, ^***^ *P* < 0.001 as determined by ANOVA.

Previous studies have shown that the administration of several tissue‐derived stem/progenitor cells (e.g., bone marrow, fat, iPSCs, and so on) to the intervertebral disc can attenuate IDD and/or promote repair of the NP ^[^ ^11^, ^15^ ^]^ *–*.^[^ ^17^ ^]^ We generated a puncture‐induced injury mouse model in the caudal spine and administered FACS‐sorted Shh‐Cre;Ai9^+^UTS2R^+/−^ cells (embedded in Matrigel) to punctured discs to investigate whether the supplementation of resident progenitor cells to NP was a potential therapeutic strategy (**Figure** **7**A). The puncture injury was established at coccygeal‐level discs (Co4–5, Co5–6, and Co6–7), while Co7‐8 was left intact as the non‐operated control (Figure 7A). The puncture decreased the disc height index (DHI) of Co4‐5 to Co6‐7, while the administration of UTS2R^+^ NP cells significantly attenuated the degeneration process compared with that of UTS2R^−^ NP cells and in the vehicle groups (Figure 7B,C), indicating the potential effect of UTS2R^+^ cells in attenuating injury‐induced IDD. Safranin O/Fast Green staining showed that puncture‐induced degeneration completely diminished the integrated structure of the disc including NP and AF and markedly reduced the proteoglycan content. UTS2R^−^ NP cell administration had no noticeable effect in attenuating IDD, although the proteoglycan content slightly increased compared with that in the vehicle groups (Figure 7D,E). In contrast, UTS2R^+^ NP administration maintained the integrity of NP structure and completely attenuated the loss of proteoglycan (Figure 7D,E), indicating the potential therapeutic effect of these cells in attenuating IDD.

### 2.8 Tenascin‐C was the Potential Niche of ProNPs and Modulated its Maintenance

**Figure 8:**
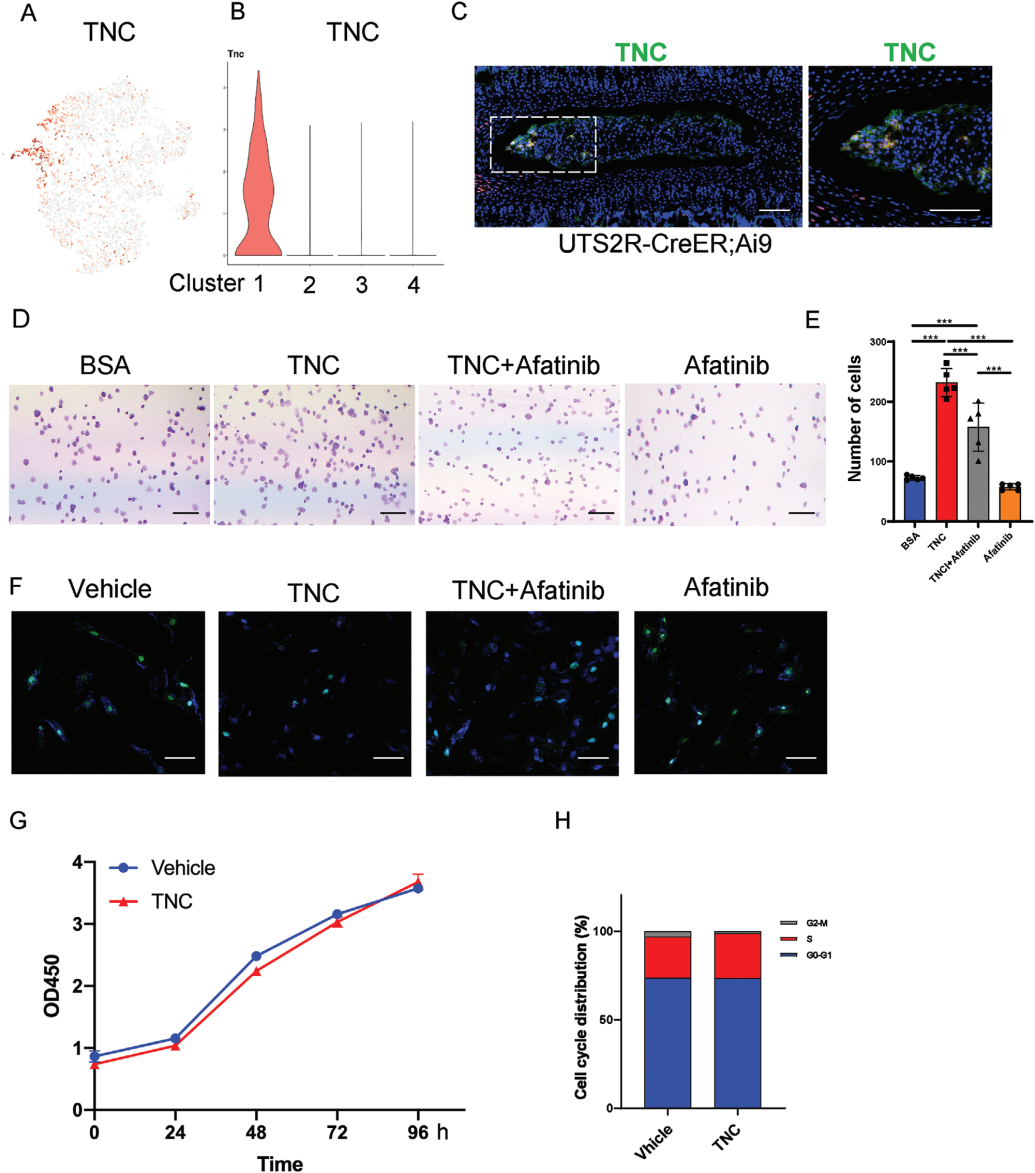
Tenascin‐C was expressed in the ProNPs niche and maintained homeostasis. A,B) Dot plot (A) and representative violin plot (B) showing the expression of TNC in Clusters 1–4. C) Representative immunofluorescence imaging of Uts2r‐CreER;Ai9^+^ cells (red) and TNC (green) in postnatal 1‐month‐old Uts2r‐CreER;Ai9/+ mice traced from P3. The right image shows high magnification of the indicated area from the left image (*n* = 3 mice per group). Scale bars, 100 µm. D,E) Cell adhesion analysis (D) and quantification (E) of rat UTS2R^+^ NP cells. FACS‐sorted UTS2R^+^ NP cells cultured on a plate pre‐coated with BSA (control), 500 ng mL^−1^ TNC, 500 ng mL^−1^ TNC + 10 × 10^−6^ m Afatinib, and 10 × 10^−6^ m Afatinib alone for 24 h. F) TUNEL staining of rat UTS2R^+^ NP cells cultured with a vehicle, 500 ng mL^−1^ TNC, 500 ng mL^−1^ TNC + 10 × 10^−6^ m Afatinib, and 10 × 10^−6^ m Afatinib under serum‐free‐induced apoptosis for 12 h. G,H) CCK8 (G) and Cell cycle analysis (H) of rat UTS2R^+^ NP cells cultured with or without 500 ng mL^−1^ TNC. *n* = 3. Data are presented as mean ± standard deviation. ^*^ *P* < 0.05, ^**^ *P* < 0.01, ^***^ *P* < 0.001 as determined by ANOVA.

The tracing of postnatal ProNPs previously showed that these cells preferentially resided in the peripheral region of the NP (Figure 4B,E). We examined the expression of various ECM molecules among NP cells to further investigate this potential ProNP niche. Tenascin‐C (TNC), an ECM glycoprotein that has been reported to maintain the niche of neural stem cells and modulate their proliferation,^[^ ^18^, ^19^, ^20^ ^]^ was specifically expressed in the ProNP cluster (**Figure** **8**A,B). The immunostaining of TNC in lumbar spine sections of 1‐month‐old Uts2r‐CreER;Ai9 mice showed that Uts2r‐CreER;Ai9^+^ NP cells were preferentially embedded in the TNC‐expressing region (Figure 8C), indicating that this was indeed a potential niche of ProNPs. We sorted rat UTS2R^+^ ProNPs and treated them with recombinant TNC, which contained most of the EGF‐like domain, a few fibronectin‐type III‐like domains, and a terminal fibrinogen globe, to investigate the physiological effect of TNC on ProNPs.^[^ ^21^, ^22^ ^]^ UTS2R^+^ ProNPs were cultured with TNC in the presence or absence of EGFR inhibitor (Afatinib), or EGFR inhibitor alone (Figure 8D,E). The cell adhesion analysis showed that TNC significantly induced the adhesion of UTS2R^+^ ProNPs, while Afatinib markedly attenuated the aforementioned effect of TNC (Figure 8D,E). In addition, we induced the apoptosis of UTS2R^+^ ProNPs in serum‐free culture. TUNEL staining showed that TNC markedly inhibited cell apoptosis, while Afatinib markedly attenuated the anti‐apoptotic effect of TNC. Of note, although Afatinib inhibited the proliferation of UTS2R^+^ ProNPs, TNC did not modulate cell proliferation (Figure 8G,H), indicating that TNC served as the potential niche of ProNPs modulating its maintenance and keeping it quiescent.

### 2.9 UTS2R^+^ ProNPs Combined with Tenascin‐C was a Promising Therapeutic Intervention to Attenuate IDD

**Figure 9:**
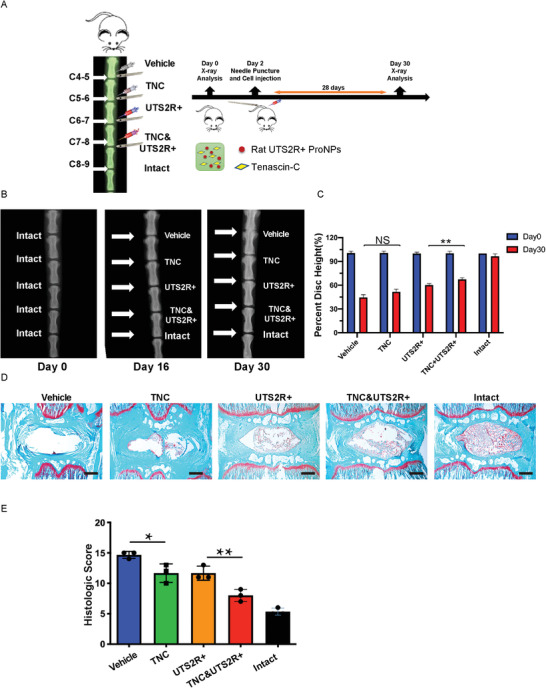
UTS2R^+^ ProNPs combined with tenascin‐C was a promising therapeutic to attenuate IDD. A) Diagram of the experiment outline displaying the time points and procedure of establishing the caudal spine injury model and transplanting rat UTS2R^+^ NP cells embedded with/without tenascin‐C (TNC). B,C) X‐ray scan and quantification revealing the caudal disc space and height between vehicle‐treated, TNC‐treated, UTS2R^+^ NP–transplanted, UTS2R^+^ NP cells embedded with TNC transplanted, and intact groups. D,E) Safranin O and Fast Green staining (D) and the Histologic Score (E) revealing the progress of injury‐induced IDD between vehicle‐treated, TNC‐treated, UTS2R^+^ NP–transplanted, UTS2R^+^ NP cells embedded with TNC transplanted, and intact groups. *n* = 3. Data are presented as mean ± standard deviation. ^*^ *P* < 0.05;^**^ *P* < 0.01; N.S., not significant as determined by ANOVA.

The harsh local environment of NP cells presents a challenge to cellular treatments for IDD, as supplemented cells often fail to thrive. To circumvent this issue, we investigated a combination of resident ProNPs with their own niche ECM as a therapeutic strategy. We established the puncture‐induced tail disc injury model further in 6‐week‐old SD rats and administered FACS‐sorted rat UTS2R^+^ ProNPs (embedded with or without TNC in Matrigel) to punctured discs (**Figure** **9**A). The puncture injury was established at coccygeal‐level discs (Co4–5, Co5–6, Co6–7, and Co7‐8), while Co8‐9 was left intact as the non‐operated control (Figure 9A). The puncture decreased the DHI of Co4‐5 to Co7‐8, while the administration of UTS2R^+^ NP cells embedded with TNC significantly attenuated the degeneration process compared with UTS2R^+^ NP cells, TNC alone, or vehicle control (Figure 9B,C). Safranin O/Fast Green staining showed that UTS2R^+^ NP cells embedded with TNC significantly attenuated the degeneration process compared with the rest groups (Figure 9D,E), indicating that combining UTS2R^+^ ProNPs with its potential niche factor‐TNC, was a promising potential therapeutic strategy in attenuating IDD.

## 3 Discussion

**Figure 10:**
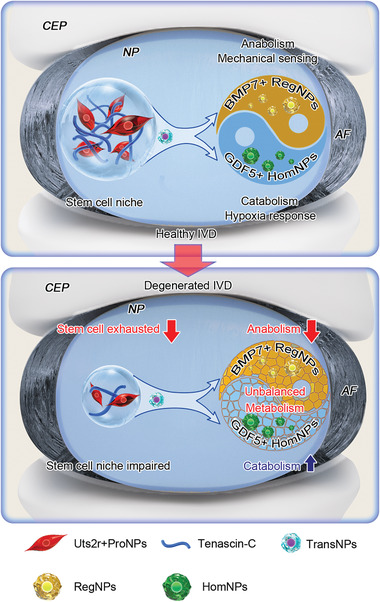
Diagram showing the role and function of NP cell subpopulations during IVD homeostasis and degeneration.

The origin and heterogeneity of cells in the nucleus pulposus, which are believed to be derived from the embryonic notochord with high homogeneity, have been debatable for a long time.^[^ ^23^, ^24^ ^]^ We previously discovered that LepR^+^ cells represented a distinct subpopulation of notochord‐derived cells, and determined that postnatal NP cells were heterogeneous and might contain potential stem/progenitor cells.^[^ ^13^ ^]^ The operative limitations and the difficulties in isolating pure cell population murine disc tissue have limited NP research in the last decades; several sequencing analyses of RNA, proteins, or single cells have been based on mixed IVD tissue. However, pure murine NP cells can be isolated using NP‐specific fluorescence reporter mice (Shh‐Cre and Noto‐Cre) and FACS sorting strategies, enabling us to fully characterize the NP cell population. This study was novel in isolating pure NP cells using Shh‐Cre;Ai9 fluorescence reporter mice. Subsequently, the scRNA‐seq analysis revealed the heterogeneity and diverse roles for postnatal NP subpopulations without contamination by AF cells during normal homeostasis. Previous studies identified Tie2^+^GD2^+^ cells in the NP as NP progenitor cells; several other studies also indicated via in vitro analysis that NP cells might contain progenitors.^[^ ^25^, ^26^, ^27^, ^28^ ^]^ However, the source and origin of NP progenitors have long been disputed, especially during postnatal stages, which has raised the possibility of IVD regeneration/repair. In this study, we discovered four distinct subpopulations in murine NP cells: ProNPs, TransNPs, RegNPs, and HomNPs. We confirmed the existence of postnatal ProNPs combining scRNA‐seq, lineage tracing (Uts2r‐CreER;Ai9 mice), and FACS analysis (gating Shh‐Ai9^+^UTS2R^+^ ProNPs and analyzing for stem/progenitor cell properties). These cells were located at the origin of the trajectory process, resided in their own niche (TNC‐enriched ECM), displayed self‐proliferation and multi‐lineage differentiation capacities in vitro, gave rise to functional NP cells in vivo, and attenuated IDD progression after transplantation (**Figure** **10**).

NP tissue in the intervertebral disc is an immunologically privileged tissue with avascular and non‐innervated properties. The NP tissue residing in the intermediate region was originally characterized as a whole by its high proteoglycan‐to‐collagen content,^[^ ^29^ ^]^ sensitivity to mechanical stimuli,^[^ ^30^, ^31^ ^]^ and modulation by the circadian rhythm.^[^ ^32^ ^]^ During IDD, NP cells gradually transition to chondrocyte‐like cells with an unbalanced secretion of metabolic and catabolic ECM enzymes. In the meantime, vessels and nerve fibers grow into the NP and open the “window” to inflammatory and immunological cascades, which may be the leading causes of discogenic back pain.^[^ ^33^ ^]^ In this study, we also identified functional RegNPs and HomNPs, which played distinct roles in maintaining IVD homeostasis. RegNPs were metabolically active and mechanically sensitive, which preferentially expressed anabolic metabolism genes, highly expressed Ngf, and had pro‐angiogenic and pro‐inflammatory properties. HomNPs, which sensitively responded to the hypoxic environment, preferentially expressed catabolic metabolism genes, possessed anti‐inflammatory and anti‐angiogenesis potency, and likely acted as a counterweight to RegNPs. Of note, the cell communication analysis revealed that HomNPs were primarily modulated by RegNPs, indicating a possible inherent regulation of homeostasis within NP subpopulations. Under physiological conditions, RegNPs sense mechanical signals and are involved in major metabolic activities. HomNPs respond to the hypoxia microenvironment and play anti‐inflammatory and anti‐angiogenic roles, which together maintain disc homeostasis in the steady state. At the onset of IDD, RegNPs start expressing pro‐angiogenic and pro‐inflammatory genes, which subsequently induces vessel growth into NP tissue and triggers the immunologic cascade, potentially leading to the onset and progression of discogenic back pain. Moreover, IDD data from mice and patients showed that the number of HomNPs increased dramatically under IDD conditions, indicating that HomNPs might be driven to undergo fibrosis when disc homeostasis broke down and the inflammatory cascade was activated. Comprehensive analyses, including fate mapping and functional and molecular signaling targeting the interactions between RegNPs and HomNPs are needed for future research.

Of note, Zhang et al. showed a promising strategy of inducing human pluripotent stem cells to differentiate into notochord‐like and nucleus pulposus‐like cells using a defined protocol.^[^ ^34^ ^]^ We can definitely advance the development of pre‐clinical translation using iPSCs or NP subpopulations by combining the potential TFs (with downstream targets) and therapeutic targets in either ProNPs or functional RegNPs and HomNPs from our findings.

Stem/Progenitor cells occupy a specific stem cell niche, which is a specialized and unique microenvironment where stem cells reside in a quiescent state. The stem cell niche is intricate and may consist of various cell types and multiple extracellular factors. ECM proteins are involved in stem cell maintenance and renewal and are recently regarded as important components of various stem cell niches.^[^ ^35^, ^36^, ^37^ ^]^ Not surprisingly, tenascin‐C, a highly conserved ECM glycoprotein, is an important extracellular niche factor found in many stem cell niches. These include neural, follicle, and hematopoietic stem cell niches, and even the metastatic niche in cancer progression, indicating the important role of TNC during tissue homeostasis and disease.^[^ ^18^, ^38^, ^39^ ^]^

In conclusion, we reported the first scRNA‐seq analysis of murine spine combined with NP cell lineage tracing, FACS analysis, and murine/human IDD sample analysis. These assays revealed the heterogeneity and diverse roles for NP cell subpopulations during disc homeostasis and degeneration, suggesting potential diagnostic and therapeutic strategies for IDD and its related symptoms, especially discogenic back pain.

## 4 Experimental Section

### Patient Samples

The nucleus pulposus specimens were obtained from 16 patients (6 male and 10 female; mean age 44.38 ± 35.62 years) with degenerative disc disease or scoliosis. The degree of IDD was assessed according to the modified Pfirrmann grading system by magnetic resonance imaging (MRI).^[^ ^40^ ^]^ All the information on Grade II (*n* = 5), Grade III (*n* = 3), Grade IV (*n* = 3), and Grade V (*n* = 5) samples are shown in Table S2. The ethics approval was obtained from the Institutional Review Board of Xiing Hospital of the Fourth Military Medical University (KY20203146‐1), and informed consent was obtained from each donor. The study was conducted according to the Code of Ethics of the World Medical Association (Declaration of Helsinki).

### Animals and Treatment

Rosa26‐loxp‐stop‐loxp‐tdTomato (Rosa26‐Ai9) reporter mice were obtained as previously described.^[^ ^41^ ^]^ Shh‐Cre mice were a gift from Prof. Zhaocai Zhou (State Key Laboratory of Genetic Engineering, Department of Cell and Developmental Biology, School of Life Sciences, Zhongshan Hospital, Fudan University, Shanghai, 200438, China). All mice analyzed were maintained on the C57BL/6 background. Sprague–Dawley (SD) rats were purchased from Shanghai Lingchang Biotech Limited Company (Shanghai, China); they were housed in individually ventilated cages, two per cage, and acclimated to the vivarium for at least 3 days before experiments. All animals were housed in specific pathogen–free conditions in the institutional animal facility of the Shanghai Institute of Biochemistry and Cell Biology, Chinese Academy of Sciences. The genotypes of the mice were determined by PCR analyses of genomic DNA extracted from mouse‐tail snips using the following primers:

Shh‐Cre‐forward: 5′‐GATGTGTTCCGTTACCAGCGA‐3′
Shh‐Cre‐reverse: 5′‐ATGAACTTCAGGGTCAGCTTGC‐3′
Uts2r‐CreER‐forward: 5′‐TGCCTGGCTCAGCATTTCTC‐3′
Uts2r‐CreER‐reverse: 5′‐GCACAATGTAGCTGTCAGACACAC‐3′
R26R‐tdTomato‐forward‐1: 5′‐AAGGGAGCTGCAGTGGAGTA‐3’
R26R‐tdTomato‐forward‐2: 5′‐CCGAAAATCTGTGGGAAGTC‐3′
R26R‐tdTomato‐reverse1: 5′‐ GGCATTAAAGCAGCGTATCC‐3′
R26R‐tdTomato‐reverse2: 5’‐CTGTTCCTGTACGGCATGG‐3’.

### Ethics Statement

All relevant ethical regulations were compiled for animal testing and research. All animal experiments were performed in the Animal Facility of Shanghai Institute of Biochemistry and Cell Biology and according to the protocol (approval number: SIBCB‐NAF‐14‐001‐S350‐019) authorized by the Animal Care and Use Committee of Shanghai Institute of Biochemistry and Cell Biology, Chinese Academy of Sciences.

### Single‐Cell RNA Sequencing

A BD Rhapsody single‐cell analysis system was used to capture the transcriptomic information of the Shh‐Cre;Ai9^+^ NP single cells. Single‐cell RNA sequencing was performed by NovelBio Bio‐Pharm Technology Co., Ltd. The single‐cell capture was achieved by the random distribution of a single‐cell suspension across >200 000 microwells through a limited dilution approach. Beads with oligonucleotide barcodes were added to saturation so that a bead was paired with a cell in a microwell. The cells were lysed in the microwell to hybridize mRNA molecules to barcoded capture oligos on the beads. The beads were collected in a single tube for reverse transcription and *Exo*I digestion. Upon cDNA synthesis, each cDNA molecule was tagged on the 5′ end (that is, the 3′ end of the mRNA transcript) with a unique molecular identifier (UMI) and cell barcode indicating its cell of origin. Whole‐transcriptome libraries were prepared using the BD Rhapsody single‐cell whole‐transcriptome amplification (WTA) workflow, including random priming and extension (RPE), RPE amplification PCR, and WTA index PCR. The libraries were quantified using a high‐sensitivity DNA chip (Agilent) on a Bioanalyzer 2200 and the Qubit high‐sensitivity DNA assay (Thermo Fisher Scientific). The sequencing was performed with an Illumina sequencer (Illumina, CA, USA) on a 150‐bp paired‐end run.

### Single‐Cell RNA Statistical Analysis

The scRNA‐seq data analysis was performed with NovelBrain Cloud Analysis Platform. fastp was applied with a default parameter filtering the adaptor sequence and removed the low‐quality reads to achieve clean data.^[^ ^42^ ^]^ UMI‐tools was applied for single‐cell transcriptome analysis to identify the cell barcode whitelist.^[^ ^43^ ^]^ The UMI‐based clean data were mapped to the mouse genome (mm10 Ensemble version 92) using STAR mapping with customized parameters from the UMI‐tools standard pipeline to obtain the UMI counts of each sample.^[^ ^44^ ^]^ The cells containing more than 200 expressed genes and mitochondria UMI rate <20% passed the cell quality filtering, and mitochondria genes were removed in the expression table. Seurat package (seurat 2.3.4) was used for cell normalization and regression based on the expression table according to the UMI counts of each sample and percentage of mitochondria rate to obtain the scaled data. PCA was constructed based on the scaled data. The top 2885 highly variable genes and top 10 principals were used for t‐SNE construction and UMAP construction. The conjoint analysis was performed using the harmony algorithm to correct the batch effect between samples and re‐running t‐SNE construction and UMAP construction. The unsupervised cell cluster result based on the PCA top 10 principals was acquired using the graph‐based cluster method (resolution = 0.8) and the marker genes were determined by the FindAllMarkers function with the Wilcoxon rank‐sum test algorithm under the following criteria: lnFC >0.25; *P* value <0.05; min.pct >0.1. The clusters of the same cell type were selected for t‐SNE reanalysis, graph‐based clustering, and marker analysis to identify the cell type in detail.

### Pseudotime Analysis

The single‐cell trajectory analysis using Monocle 2 (http://cole‐trapnell‐lab.github.io/monocle‐release) with DDR‐Tree and default parameter was applied. Before Monocle analysis, marker genes of the Seurat clustering result and raw expression counts of the cells that passed filtering was selected. Based on the pseudotime analysis, branch expression analysis modeling was applied for the branch fate–determined gene analysis.

### RNA Velocity

RNA velocity analysis was performed using veloyto.py, velocyto.R.^[^ ^45^ ^]^

### Cell Communication Analysis

The cell communication analysis based on the CellPhoneDB, a public repository of ligands, receptors, and their interactions was applied, to enable a systematic analysis of cell–cell communication molecules.^[^ ^46^ ^]^ The membrane, secreted, and peripheral proteins of the cluster of different time points were annotated. Significant mean and cell communication significance (*P* value <0.05) were calculated based on the interaction and the normalized cell matrix achieved by Seurat normalization.

### SCENIC Analysis

The single‐cell regulatory network inference and clustering (pySCENIC, v0.9.5) workflow,^[^ ^47^ ^]^ using the 20‐thousand motif database was applied for RcisTarget and GRNboost, to assess transcription factor regulation strength.

### QuSAGE Analysis (Gene Enrichment Analysis)

QuSAGE (2.16.1) analysis was performed to characterize the relative activation of a given gene set, such as “Angiogenesis” and “Fatty Acid Metabolism,” as previously described.^[^ ^48^ ^]^

### Differential Gene Expression Analysis

The function FindMarkers with the Wilcoxon rank‐sum test algorithm was used under the following criteria to identify differentially expressed genes among samples: lnFC >0.25; *P* value <0.05; min.pct >0.1.

### Immunocytochemistry and Immunofluorescence Analyses

Human NP tissues were fixed with 4% paraformaldehyde for 48 h, decalcified in 10% ethylenediaminetetraacetic acid (EDTA; pH 7.4) for 14 days, dehydrated, embedded in OCT, and sectioned at 10 µm. The immunofluorescence analysis of the intervertebral disc sections was performed as described previously.^[^ ^49^ ^]^ The sections were incubated with primary antibodies against UTS2R (Bioss, BS‐1806R), GDF5 (Novusbio, NBP2‐16631), CNTFRa (Santa Cruz Biotechnology Inc., SC9993), and mouse HMGCS1 (Proteintech, 17643‐ 1‐AP, 1:200) was incubated, followed by incubation with donkey anti‐rabbit, donkey anti‐mouse, and donkey anti‐goat (Molecular Probes, 1:1000) secondary antibodies. The nuclei were counterstained with DAPI (Sigma, D8417), and the slides were mounted in anti‐fade fluorescence mounting medium (Dako, S3023). The images were acquired using a confocal microscope (LSM880 Airyscan, Zeiss) under identical imaging conditions using identical acquisition parameters, and fluorescence signals were quantified with ImageJ v1.8.0.112.

### Establishment of a Lumbar Spine Instability Mouse Model (LSI)

Four‐week‐old Shh‐Cre;R26R;tdTomato male mice were used for this experiment. After anesthetizing with ketamine and xylazine, the mice were operated by resection of the Lumbar^2nd^–Lumbar^5th^ (L2–L5) spinous processes along with the supraspinous and interspinous ligaments to induce instability of lumbar spine, which was followed by previous protocol.^[^ ^30^, ^31^ ^]^ They were euthanized 4 weeks after the surgery, and the NP tissues were analyzed by immunofluorescence and flow cytometry.

### Establishment of a TS Model

Four‐week‐old Shh‐Cre;R26R;tdTomato male mice were randomly divided into two groups for TS and ground control. The tail was suspended to maintain the mice at a head‐down tilt at about 30°, with their hind limbs unable to touch the ground when the animals was fully stretching. The mice were suspended in individual plastic cages for 14 days,^[^ ^50^ ^]^ and the NP tissues were analyzed by immunofluorescence and flow cytometry.

### Disc Height Measurement and Image Analysis

X‐ray imaging of lumbar discs was performed using cabinet X‐ray imaging and irradiation systems (Faxitron Bioptic, LLC, Wheeling, IL, USA). Digital images were obtained at 45 kVp under identical imaging conditions using identical acquisition parameters. The relative heights of the lumbar IVDs were measured as reported previously.^[^ ^51^, ^52^ ^]^ The percentage disc height was calculated as the average of three measurements per disc. The percentage disc height of each group was calculated as the average height of three discs (L2–L5) from three mice.

### Flow Cytometry

The mouse intervertebral disc cells were extracted from Shh‐Cre; Rosa26^tdTomato^ mice and cut into pieces of ∼1 cm^3^ using ophthalmological scissors. Then, intervertebral disc tissues were digested for 5 min with 0.25% trypsin at 37°C. The digestions was terminated with *α*‐MEM + 10% fetal bovine serum (FBS). The cells were washed twice by centrifugation at 800 *g* for 5 min with ice‐cold DPBS (Corning, 21‐031‐CMR); Subsequently, 0.2% Type II collagenase was used to digest intervertebral disc tissues for 2 h. The digestion was again terminated with *α*‐MEM + 10% FBS. After the supernatant was removed, the pelleted cells were suspended in red blood cell lysis buffer (Miltenyi Biotec, C3702) to lyse red blood cells. After washing with PBS containing 0.04% BSA, the cell pellets were resuspended in PBS containing 0.04% BSA and re‐filtered through a 35‐µm cell strainer. Dissociated single cells were then stained with AF488 anti‐mouse UTS2R (R&D Systems, FAB9245G‐100UG), APC anti‐mouse GDF5 (ASSAYPRO, 32579‐05161), anti‐mouse CNTFRa (Santa Cruz Biotechnology, SC9993), BB700 anti‐mouse CD146 (BD Biosciences, 742 280), APC anti‐mouse CD44 (BioLegend, 559 250), BV421 anti‐mouse CD73 (BioLegend, 127 217), BV605 anti‐mouse CD90 (BioLegend, 140 317), APC anti‐mouse PDGFa/CD140a (BioLegend, 135 907), FITC anti‐mouse Sca‐1 (BioLegend, 108 106), PerCP/Cy5.5 anti‐CD31 (BioLegend, 102 420), PerCP/Cy5.5 anti‐CD45 (BioLegend, 103 132), PerCP/Cy5.5 anti‐mouse TER‐119 (BioLegend, 116 228), FITC anti‐mouse 6C3/Ly‐51 (BioLegend, 108 305), Brilliant Violet 605 anti‐mouse CD90.2 (BioLegend, 140 317), PE/Cy7 anti‐mouse CD105 (BioLegend, 120 409), APC anti‐mouse CD200 (BioLegend, 123 809), and anti‐mouse Alexa Fluor 488 (Molecular Probes, A21202, 1:1000). The flow cytometric analysis was performed with a Beckman Coulter CytoFLEX LX after washing twice by centrifugation at 600 *g* for 5 min with ice‐cold DPBS + 2% FBS. The flow cytometry data were analyzed using FlowJo X 10.0.7r2.

### Real‐Time RT‐PCR Analysis

RT‐PCR was performed as described previously.^[^ ^50^ ^]^ Total RNA was extracted from FACS‐sorted NP cells of Shh‐Cre;R26R;tdTomato mice using TRIzol (Sigma, T9424) and reverse transcribed into cDNA using TaKaRa PrimeScript Reverse Transcriptase (TaKaRa, RR037A). The real‐time RT‐PCR reaction was performed with the BioRad CFX96 system. The gene expression was determined relative to a housekeeping gene (HPRT) using the ΔΔC_t_ method.

The primer sets used were as follows:

Krt8‐forward: 5’‐CTCAAAGGCCAGAGGGCATC‐3’;
Krt8‐reverse: 5’‐TTAATGGCCATCTCCCCACG‐3’;
Krt18‐forward: 5’‐CAGGGACTGGAGTCATTA‐3’;
Krt18‐reverse: 5’‐GCATTGTCCACAGAACTT‐3’;
Krt19‐forward: 5’‐CTTCCGAACCAAGTTTGAGAC‐3’;
Krt19‐reverse: 5’‐AGCGTACTGATTTCCTCCTC‐3’;
CD24‐forward: 5’‐ACCCACGCAGATTTACTGCAA‐3’;
CD24‐reverse: 5’‐CCCCTCTGGTGGTAGCGTTA‐3’;
T‐forward: 5’‐GCTTCAAGGAGCTAACTAACGAG‐3’;
T‐reverse: 5’‐CCAGCAAGAAAGAGTACATGGC‐3’;
Car3‐forward: 5’‐TGACAGGTCTATGCTGAGGGG‐3’;
Car3‐reverse: 5’‐CAGCGTATTTTACTCCGTCCAC‐3’.

### Characterization of UTS2R^+^ ProNPs

UTS2R^+^ ProNPs were harvested from the NP tissue of 1‐month‐old Shh‐Cre;R26R‐tdTomato mice. Shh‐tdTomato^+^UTS2R^+^ cells were sorted by FACS. For CFU‐F assays with sorted cells, cells were sorted directly into culture at a density of 10 cells cm^−2^ in 6‐well plates in 3 mL of *α*‐MEM supplemented with glutamine (2 × 10^−3^ m), penicillin (100 U mL^−1^), streptomycin sulfate (100 µg mL^−1^), and 20% lot‐selected fetal bovine serum (FBS), ensuring that colonies would form at clonal density to allow counting. After 2–3 h of adhesion, unattached cells were removed. On day 10, the cultures with 0.5% crystal violet was fixed and stained. The colonies that contained ≥50 cells were counted.

For osteogenic differentiation, cells were seeded at a density of 5 × 10^3^ cm^−2^ with *α*‐MEM supplemented with 10% FBS, 0.1 × 10^−3^ m dexamethasone, 10 × 10^−3^ m b‐glycerol phosphate, and 50 × 10^−3^ m ascorbate‐2‐phosphate. After 3 weeks of differentiation, the mineralization capacity of the cells was evaluated by Alizarin red staining. For adipogenic differentiation, cells were seeded at a density of 5 × 10^3^ cm^−2^ with *α*‐MEM supplemented with 10% FBS, 1 × 10^−3^ m dexamethasone, 0.5 × 10^−3^ m 3‐isobutyl‐1‐methylxanthine, and 10 ng mL^−1^ of insulin for 2 wks. Lipid accumulation was identified by oil red O staining. For chondrogenic differentiation, cells (1 × 10^5^) were seeded in polypropylene tubes with high‐glucose Dulbecco's Modified Eagle Medium (Thermo Fisher Scientific) supplemented with 0.1 × 10^−3^ m dexamethasone, 1% insulin‐transferrin‐sodium selenite mix, 50 × 10^−3^ m ascorbate–2‐phosphate, 1 × 10^−3^ m sodium pyruvate, 50 µg mL^−1^ of proline, and 20 ng mL^−1^ of TGF‐*β*3 for 3 weeks in culture, the chondrogenesis ability was measured by Alcian Blue staining.

### Cell Proliferation Assay

For cell proliferation assays, 1000 NP cells were seeded in 96‐well plates (Thermo Fisher Scientific) and cultured for indicated days. At specified time points (days 0–4), 10 µL CCK‐8 cell counting Kit (Vazyme) solution was added to each well, and the cells were incubated at 37°C for 3 h. Next, absorbance was measured in single‐wavelength mode (450 nm) using a Synergy NEO HTS Multi‐Mode Microplate Reader (BioTek).

### Statistical Analysis

Data were presented as mean ± standard deviation of at least three independent experiments. Unpaired, two‐tailed Student *t* tests were used for comparisons between the two groups. For multiple comparisons, one‐way analysis of variance (ANOVA) with Bonferroni post hoc test was applied. All data were normally distributed and had similar variations between the groups. GraphPad Prism 8 was used for all statistical analyses, and *P* values less than 0.05 were considered to indicate statistically significant differences.

## Conflict of Interest

The authors declare no conflict of interest.

## Author Contributions

W.Z., Z.L., B.G. and B.J. conceived the study and designed the experiments. B.G. and B.J. performed most of the research. Z.Q.X. and Z.L. helped perform the research, provided reagents and valuable comments and helped to analyze the data. B.G., B.J., Z.L., and W.Z. analyzed data and wrote the paper. All authors discussed and commented on the manuscript.

## Supporting information

Supporting Information

Supporting Information

Supporting Information

## Data Availability

The data that support the findings of this study are available from the corresponding author upon reasonable request.
