# Supplementary material for: Discovery and Application of Postnatal Nucleus Pulposus Progenitors Essential for Intervertebral Disc Homeostasis and Degeneration
Source: Adv Sci (Weinh). 2022 Feb 23;9(13):2104888. doi: 10.1002/advs.202104888 (PMC9069184; doi:10.1002/advs.202104888)
Supplement: Supplementary file 1 — Supporting Information [file ADVS-9-2104888-s002.pdf]

ADVANCED SCIENCE
Open Access

Supporting Information

for *Adv. Sci.*, DOI 10.1002/advs.202104888

Discovery and Application of Postnatal Nucleus Pulposus Progenitors Essential for Intervertebral Disc Homeostasis and Degeneration

Bo Gao, Bo Jiang, Wenhui Xing, Zaiqi Xie, Zhuojing Luo and Weiguo Zou**

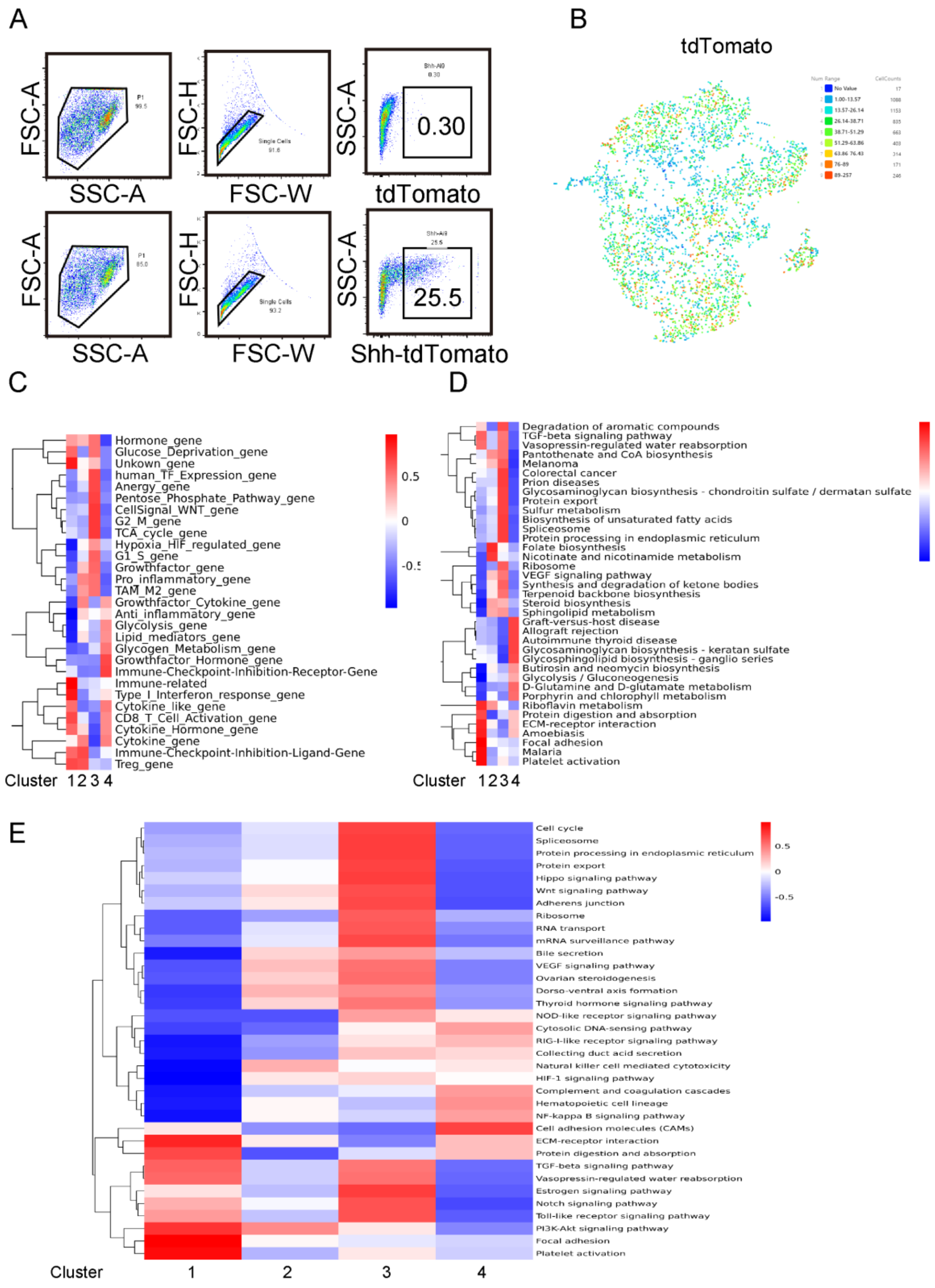

Supplementary Figure 1. FACS gating strategy of purified NP cells and the

representative KEGG predicted functions of NP subpopulations. (A) FACS analysis of gating Shh-Cre;R26R$^{tdTomato+}$ NP cells. (B) t-SNE plot of tdTomato expression gene distribution. (C–E) KEGG analysis revealing the differentially expressed genes (C), certain functions (D), and pathways (E) for each cluster.

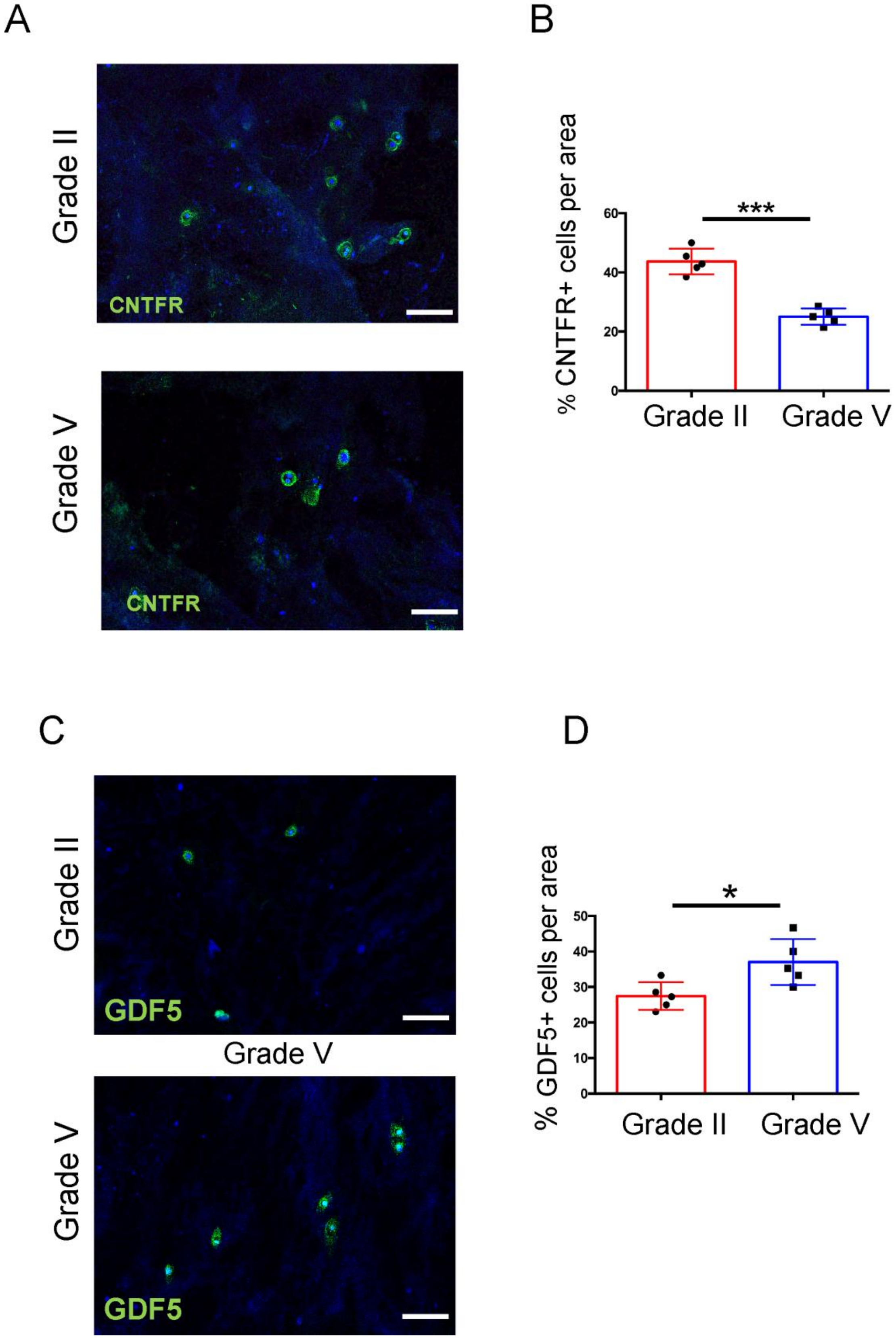
A
B
Grade II
CNTFR
Grade V
CNTFR
% CNTFR+ cells per area
60
40
20
0
***
Grade II
Grade V
C
D
Grade II
GDF5
Grade V
Grade V
GDF5
% GDF5+ cells per area
50
40
30
20
10
0
*
Grade II
Grade V

Supplementary Figure 2. Outcomes of RegNPs and HomNPs during intervertebral disc degeneration. (A–D) Immunofluorescence staining and representative quantification data of $CNTFR^+$ cells (A and B) and $GDF5^+$ cells (C and D) in human NP specimens at different degeneration levels (n=5). Data are presented as mean ± standard deviation. $^{*}P < 0.05$, $^{**}P < 0.01$ as determined by two-tailed Student *t* tests.

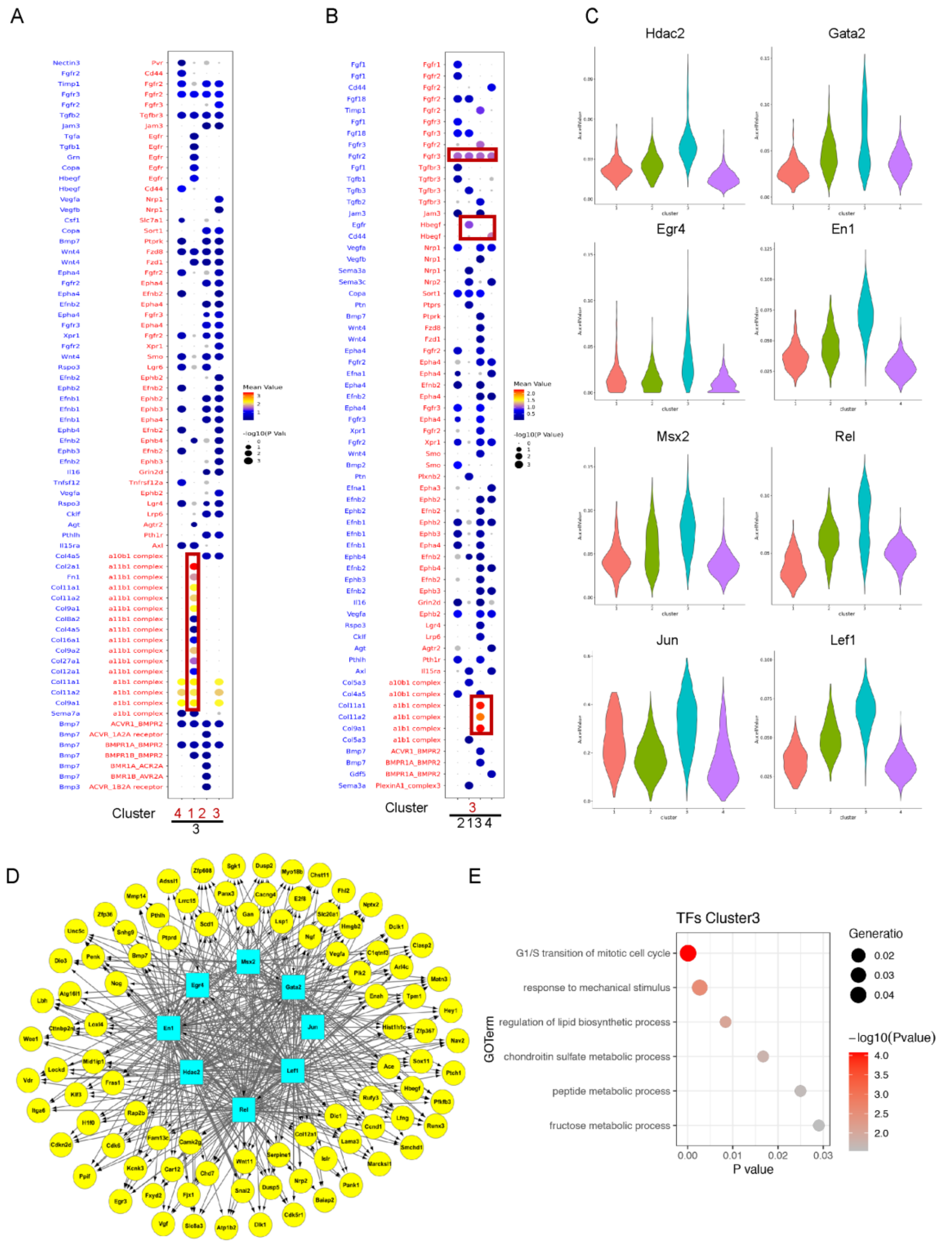

Supplementary Figure 3. Cell–cell communication and transcription factors of RegNPs. (A and B) Bubble plots of cell–cell communication showing potential interactions between RegNPs [as the ligand (A), as the receptor (B)] and other clusters. (C) Violin plots of specific transcription factors of RegNPs. (D) Predicted

downstream targets of specific RegNP transcription factors. (E) Representation analysis of GO categories showing different functions for TFs of RegNPs.

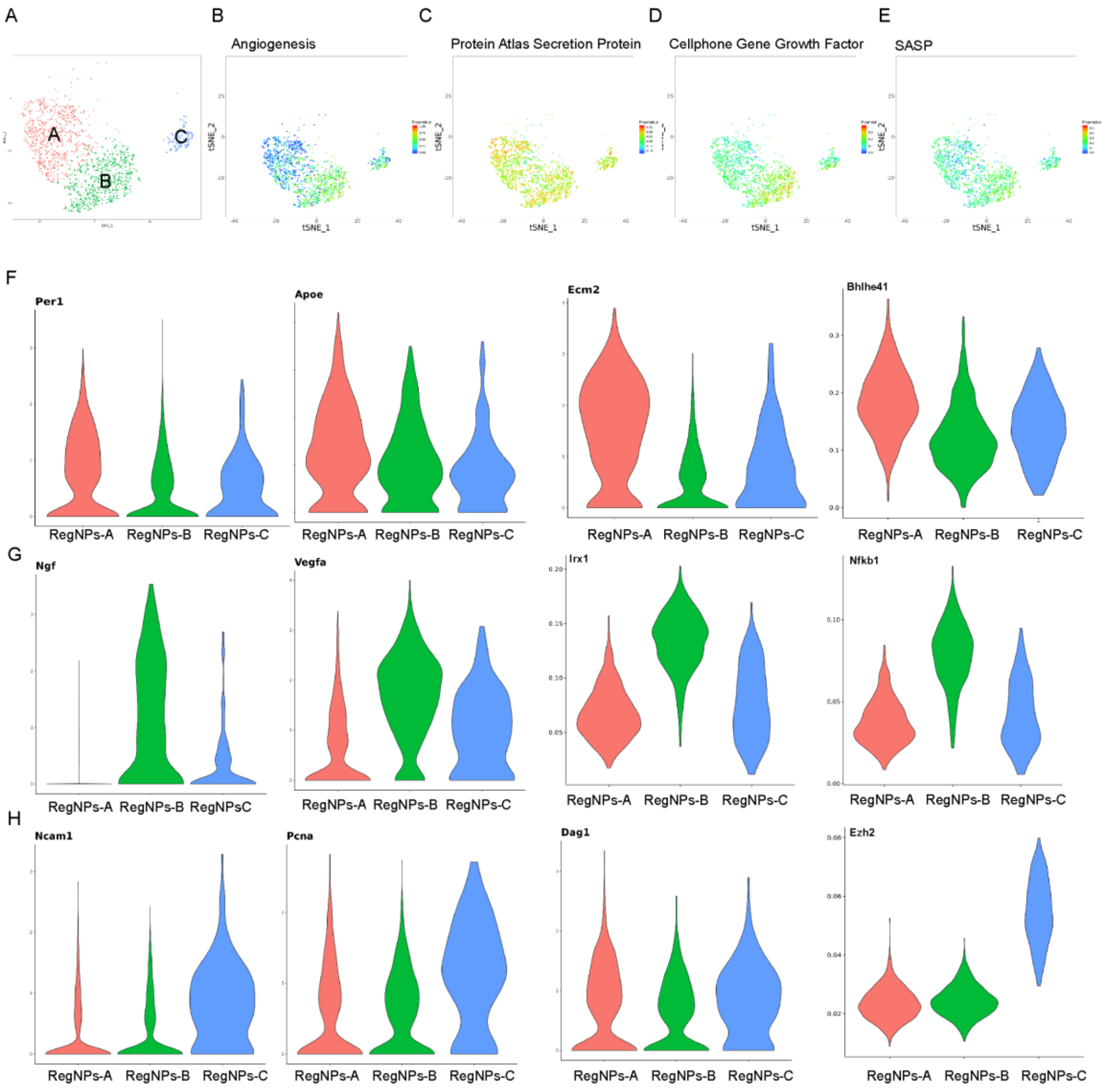

Supplementary Figure 4. RegNPs contain 3 subpopulations with distinct characteristics. (A) Representative image of t-SNE analysis according to subpopulations for RegNPs single-cell transcriptomes; (B-E) Representative t-SNE plot showing gene expression pattern related to "Angiogenesis", "Protein Atlas Secretion Protein", "Cellphone Gene Growth Factor" and "SASP"; (F-H) Representative violin plot showing the expression of Ngf, Vegfa, Irx1, Nfkb1 (F), Opn, Apoe, Ecm2, Bhlhe41 (G), Ncam1, Pcna, Dag1 and Ezh2 (H).

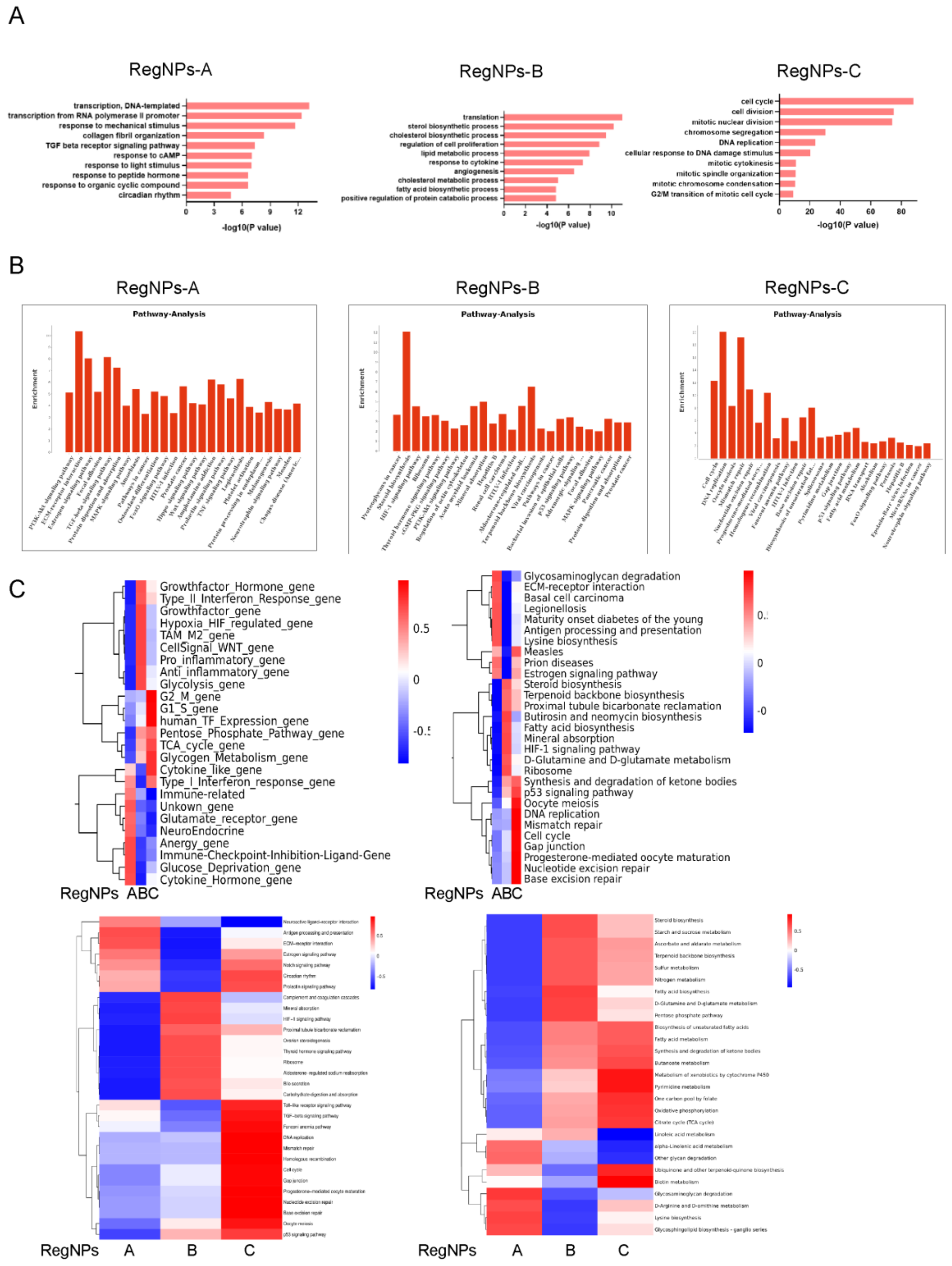

Supplementary Figure 5. Respective functions of RegNPs subpopulations. (A) Representation analysis of GO categories showing different functions for RegNPs-A,

RegNPs-B and RegNPs-C; (B) Representation analysis of KEGG categories showing different functions for RegNPs-A, RegNPs-B and RegNPs-C; (C) KEGG analysis revealing differentially expressed genes, functions and pathways for RegNPs-A, RegNPs-B and RegNPs-C.

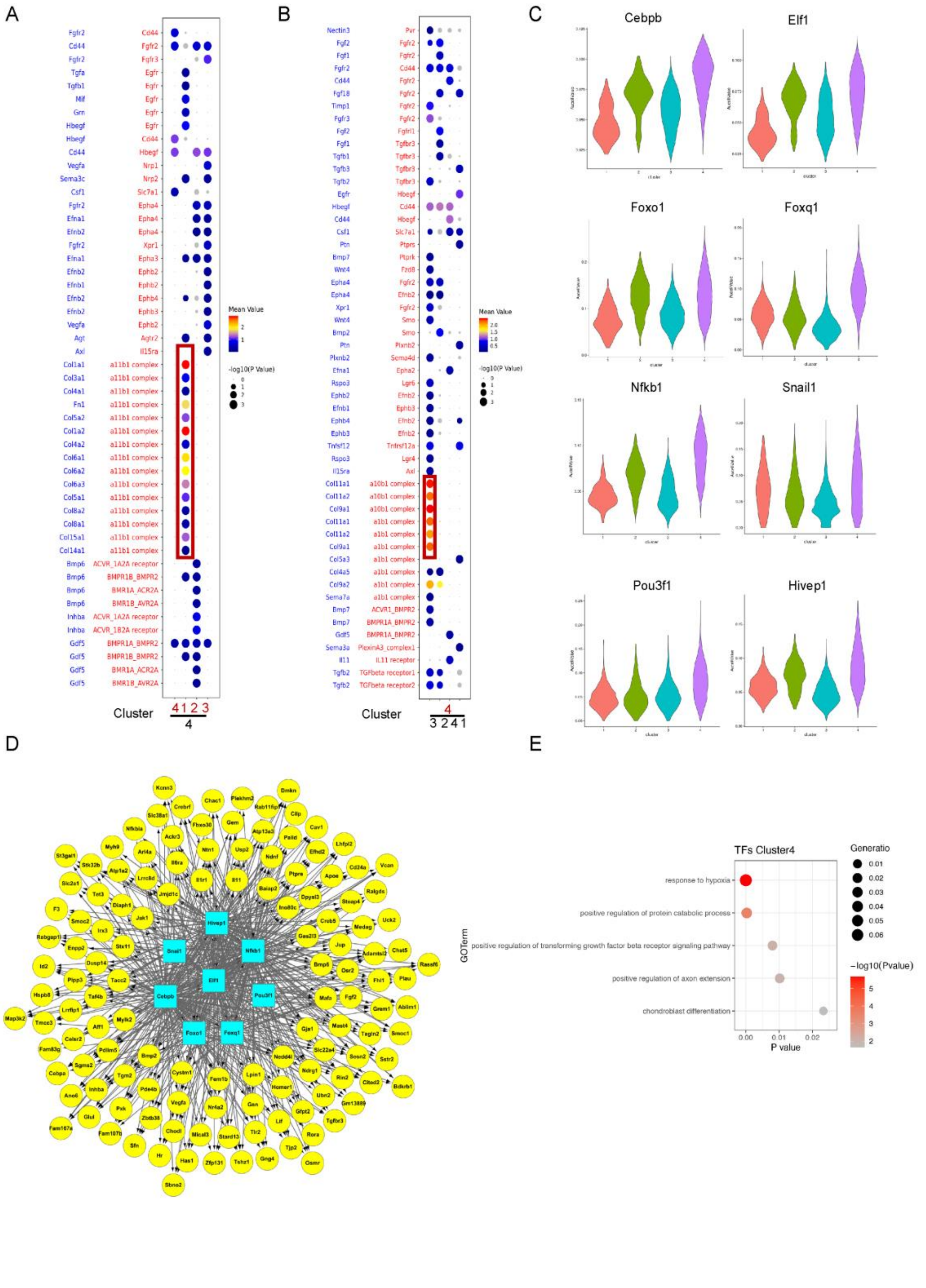

Supplementary Figure 6. Cell–cell communication and transcription factors of HomNPs. (A and B) Bubble plots of cell–cell communication showing potential

interactions between HomNPs [as the ligand (A), as the receptor (B)] and other clusters. (C) Violin plots of specific transcription factors of HomNPs. (D) Predicted downstream targets of specific HomNP transcription factors. (E) Representation analysis of GO categories showing different functions for TFs of HomNPs.

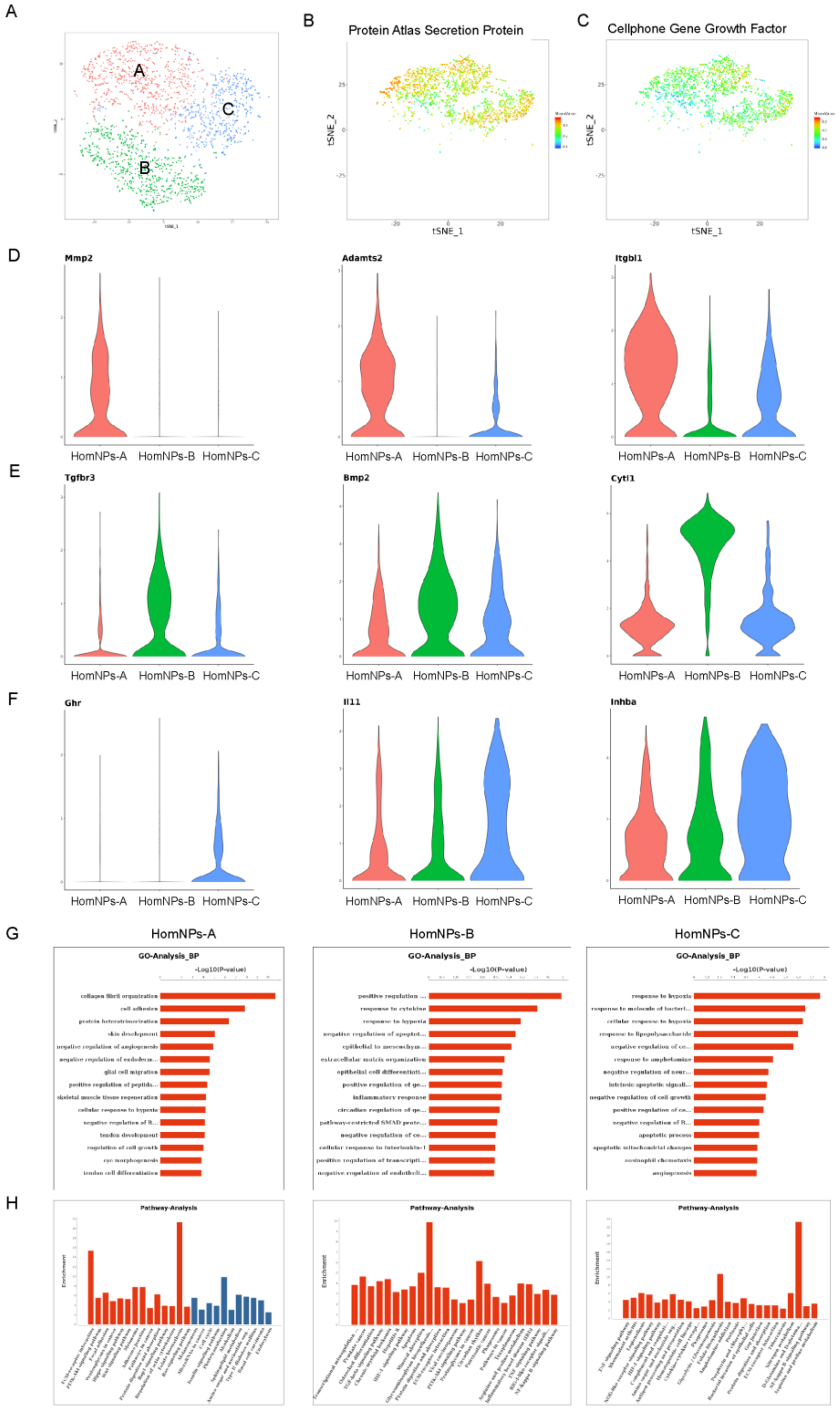

Supplementary Figure 7. HomNPs contain 3 subpopulations with distinct functions. (A) Representative image of t-SNE analysis according to subpopulations

for HomNPs single-cell transcriptomes; (B,C) Representative t-SNE plot showing gene expression pattern related to “Protein Atlas Secretion Protein” and “Cellphone Gene Growth Factor”; (D-F) Representative violin plot showing the expression of Mmp2, Adamts2, Itgbl1 (D), Tgfbr3, Bmp2, Cytl1 (E), Ghr, Il11 and Inhba (F). (G) Representation analysis of GO categories showing different functions for HomNPs-A, HomNPs-B and HomNPs-C; (H) Representation analysis of KEGG categories showing different functions for omNPs-A, HomNPs-B and HomNPs-C.

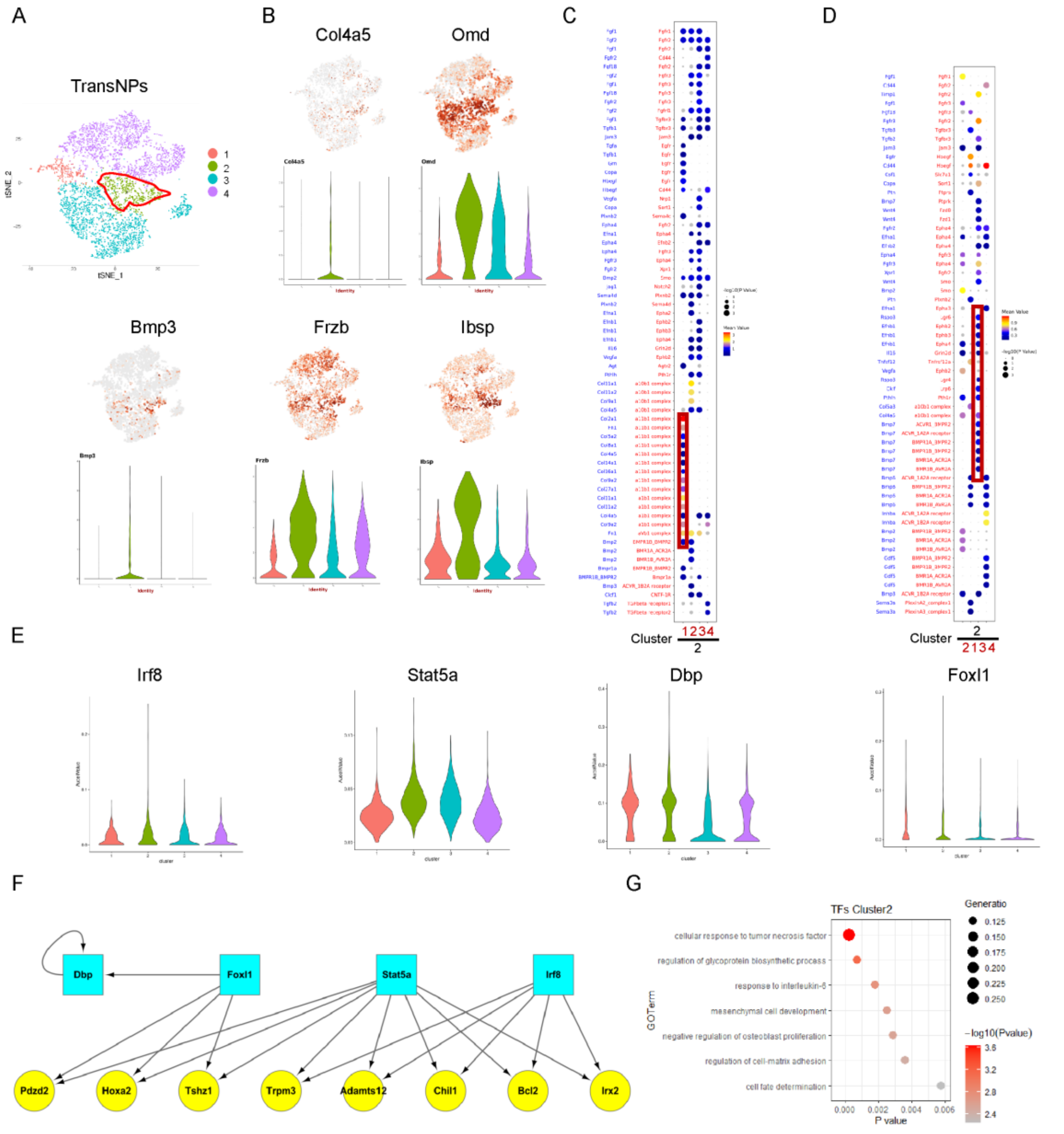

Supplementary Figure 8. Cluster 2 was a transient cell population. (A) t-SNE image displaying TransNPs distribution pattern. (B) t-SNE plots and representative

violin plots showing the expression of Col4a5, Omd, Bmp3, Frzb, and Ibsp on the t-SNE map. (C and D) Bubble plots of cell–cell communication showing potential interactions between Cluster 2 [as the ligand (C), as the receptor (D)] and other clusters. (E and F) Violin plots (E) and predicted downstream targets (F) of specific TransNP transcription factors. (G) Representation analysis of GO categories showing different functions for TFs of TransNPs.

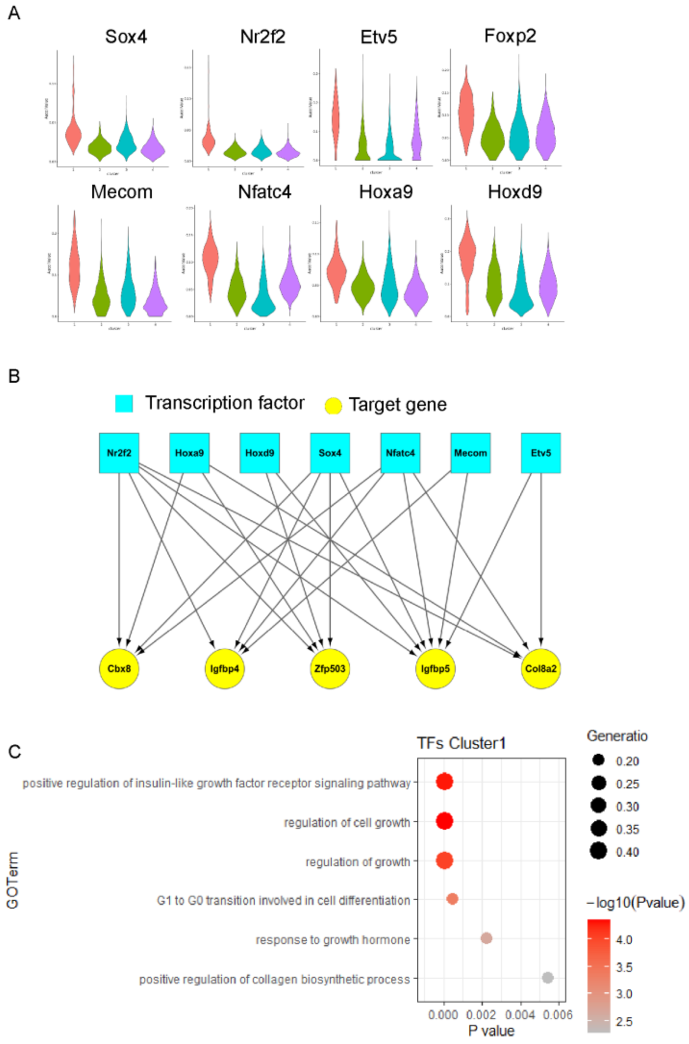

Supplementary Figure 9. Transcription factor and downstream modulation factors of ProNPs. (A and B) Violin plots (A) and predicted downstream targets (B) of specific ProNP transcription factors. (C) Representation analysis of GO categories showing different functions for TFs of ProNPs.
