# Supplementary material for: Discovery and Application of Postnatal Nucleus Pulposus Progenitors Essential for Intervertebral Disc Homeostasis and Degeneration
Source: Adv Sci (Weinh). 2022 Feb 23;9(13):2104888. doi: 10.1002/advs.202104888 (PMC9069184; doi:10.1002/advs.202104888)
Supplement: Supplementary file 3 — Supporting Information [file ADVS-9-2104888-s003.docx]

**Table S1. Demographic data of patients.**

| Patients NO. | Age | Gender | Level | Pfirrmann grading |
| --- | --- | --- | --- | --- |
| Grade II/III group | | | | |
| 1 | **6** | **M** | **L3/4** | **II** |
| 2 | **13** | **F** | **L2/3** | **II** |
| 3 | **12** | **F** | **L3/4** | **II** |
| 4 | **22** | **F** | **L5/S1** | **II** |
| 5 | **48** | **F** | **L5/S1** | **II** |
| 6 | **30** | **M** | **L5/S1** | **III** |
| 7 | **51** | **M** | **L4/5** | **III** |
| 8 | **64** | **F** | **L4/5** | **III** |
| Grade IV/V group | | | | |
| 9 | **40** | **M** | **L5/S1** | **IV** |
| 10 | **78** | **F** | **L4/5** | **IV** |
| 11 | **80** | **F** | **L4/5** | **IV** |
| 12 | **32** | **M** | **L4/5** | **V** |
| 13 | **51** | **F** | **L5/S1** | **V** |
| 14 | **54** | **F** | **L5/S1** | **V** |
| 15 | **58** | **M** | **L4/5** | **V** |
| 16 | **71** | **F** | **L4/5** | **V** |
